# Author Correction: Clonal dynamics and somatic evolution of haematopoiesis in mouse

**DOI:** 10.1038/s41586-025-09635-2

**Published:** 2025-09-19

**Authors:** Chiraag D. Kapadia, Nicholas Williams, Kevin J. Dawson, Caroline Watson, Matthew J. Yousefzadeh, Duy Le, Kudzai Nyamondo, Sreeya Kodavali, Alex Cagan, Sarah Waldvogel, Xiaoyan Zhang, Josephine De La Fuente, Daniel Leongamornlert, Emily Mitchell, Marcus A. Florez, Krzysztof Sosnowski, Rogelio Aguilar, Alejandra Martell, Anna Guzman, David Harrison, Laura J. Niedernhofer, Katherine Y. King, Peter J. Campbell, Jamie Blundell, Margaret A. Goodell, Jyoti Nangalia

**Affiliations:** 1 Department of Molecular and Cellular Biology, Baylor College of Medicine, Houston, TX USA; 2 Wellcome Sanger Institute, Wellcome Genome Campus, Hinxton, UK; 3 Early Cancer Institute, University of Cambridge, Cambridge Biomedical Campus, Cambridge, UK; 4 Institute on the Biology of Aging and Metabolism, Department of Biochemistry, Molecular Biology and Biophysics, University of Minnesota, Minneapolis, MN USA; 5 Columbia Center for Translational Immunology, Columbia Center for Human Longevity, Department of Medicine, Columbia University Medical Center, New York, NY USA; 6 Department of Pediatrics, Division of Infectious Diseases, Baylor College of Medicine, Houston, TX USA; 7 Wellcome-MRC Cambridge Stem Cell Institute, Jeffrey Cheah Biomedical Centre, Cambridge, UK; 8 Departments of Genetics, Pathology & Veterinary Medicine, University of Cambridge, Cambridge, UK; 9 Department of Haematology, University of Cambridge, Cambridge, UK; 10 The Jackson Laboratory, Bar Harbor, ME USA

**Keywords:** Evolutionary genetics, Mutation, Phylogenetics, Ageing, Haematopoietic stem cells

Correction to: *Nature* 10.1038/s41586-025-08625-8 Published online 5 March 2025

In the version of the article initially published, in the Fig. 4e *y* axis, “5 × 10^–5^” inadvertently appeared twice. The top value has now been corrected to “5 × 10^–4^” in the HTML and PDF versions of the article.

