# Supplementary material for: Clonal dynamics and somatic evolution of haematopoiesis in mouse
Source: Nature. 2025 Mar 5;641(8063):681–9. doi: 10.1038/s41586-025-08625-8 (PMC12074984; doi:10.1038/s41586-025-08625-8)
Supplement: Supplementary file 1 — Supplementary Notes 1–4, Figs. 1–14, Tables 1–3 and references. [file 41586_2025_8625_MOESM1_ESM.docx]

Supplementary Material for

**Clonal dynamics and somatic evolution of haematopoiesis in mouse**

Chiraag D. Kapadia, Nicholas Williams, Kevin J. Dawson, Caroline Watson, Matthew J. Yousefzadeh, Duy Le, Kudzai Nyamondo, Sreeya Kodavali, Alex Cagan, Sarah Waldvogel, Xiaoyan Zhang, Josephine De La Fuente, Daniel Leongamornlert, Emily Mitchell, Marcus A. Florez, Krzysztof Sosnowski, Rogelio Aguilar, Alejandra Martell, Anna Guzman, David Harrison, Laura J. Niedernhofer, Katherine Y. King, Peter J. Campbell, Jamie Blundell,
Margaret A. Goodell, Jyoti Nangalia

Table of Contents

[Supplementary Note 1: Age equivalents between mouse and human 3](#_Toc187305855)

[Supplementary Figure S1: Mouse and human survival data. 3](#_Toc187305840)

[Supplementary Note 2: Ancestral cell identity inference 4](#_Toc187305856)

[Supplementary Figure S2: Ancestral cell identity inference with no assumption of HSC or MPP hierarchy. 4](#_Toc187305841)

[Supplementary Figure S3: Ancestral cell identity inference assuming an HSC common ancestor or MPP common ancestor 5](#_Toc187305842)

[Supplementary Table S1: Log likelihood values and Akaike information Criterion assessing model fit 6](#_Toc187334836)

[Supplementary Figure S4: Transition Type Counts 7](#_Toc187305843)

[Supplementary Table S2: Cell identity transition rates per unit molecular time derived using the hidden Markov approach 7](#_Toc187334837)

[Supplementary Table S3: Cell identity transition rates per unit molecular time derived using the Stan-based Bayesian model 7](#_Toc187334838)

[Supplementary Figure S5: Cumulative Distribution of Specification Timing 8](#_Toc187305844)

[Supplementary Note 3: Quality control of targeted duplex-sequencing 9](#_Toc187305857)

[Supplementary Figure S6: Error correction strategy in targeted duplex sequencing 9](#_Toc187305845)

[Supplementary Figure S7: Sequencing coverage at targeted loci. 10](#_Toc187305846)

[Supplementary Figure S8: Duplex coverage at coding exons in Dnmt3a, Tet2, and Asxl1 11](#_Toc187305847)

[Supplementary Figure S9: Trinucleotide spectra of duplex-sequencing variants. 12](#_Toc187305848)

[Supplementary Figure S10. Native CH in biological replicate samples 13](#_Toc187305849)

[Supplementary Figure S11: True clone discovery across error rates 14](#_Toc187305850)

[Supplementary Figure S12: True clone discovery with error-free sequencing 15](#_Toc187305851)

[Supplementary Figure S13: False positive variant detection 15](#_Toc187305852)

[Supplementary Figure S14: Dilution of mutant reads and variant call concordance 16](#_Toc187305853)

[Supplementary Note 4. Inferring population size and division rates from cell phylogenies 17](#_Toc187305858)

[Supplementary note references 26](#_Toc187305859)

Supplemental Data

Supplementary Data 1: List of coding variants detected in phylogenetic trees and associated
dn/ds output table.

Supplementary Data 2: Duplex target panel and all variants detected by duplex sequencing.

# Supplementary Note 1: Age equivalents between mouse and human

We used mouse and human survival data to estimate age equivalency between species. The median lifespan of C57BL/6J laboratory mice is 28-months^10^ (published data reproduced in Supplementary Fig.S1). We retrieved 2017 life-table data from the USA and the UK compiled at the Human Mortality Database (mortality.org). Only female data was included to match the makeup of our aged mouse dataset. We took the average of the median lifespans in the UK (82.5) and USA (80.7) to estimate the female mean lifespan as 81.6 years. Lastly, we normalised mouse age by median lifespan to determine an estimated equivalent human age. The above was only performed for the aged samples. Mice reach sexual maturity earlier in lifespan relative to humans, so age-equivalency was determined by onset of reproductive maturity between species, as previously described^75^.


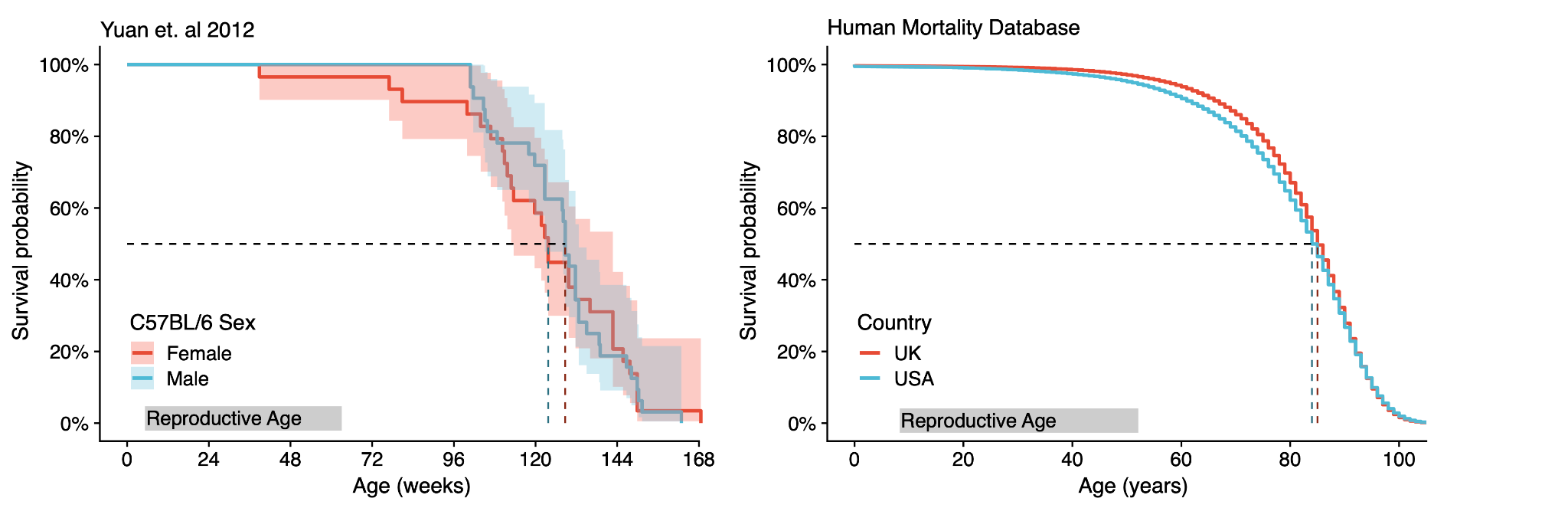


Supplementary Figure S1: Mouse and human survival data.

***Supplementary Figure S1:*** *Mouse (C57BL/6J strain) survival data (left graph) by age for males (blue) and females (red). Human survival data (right graph) by age for females in the UK (red) and USA (blue). Dashed black lines mark the age of 50% survival probability for both species. Shaded area represents 95% confidence interval. Reproductive age is highlighted by the grey box.*

# Supplementary Note 2: Ancestral cell identity inference


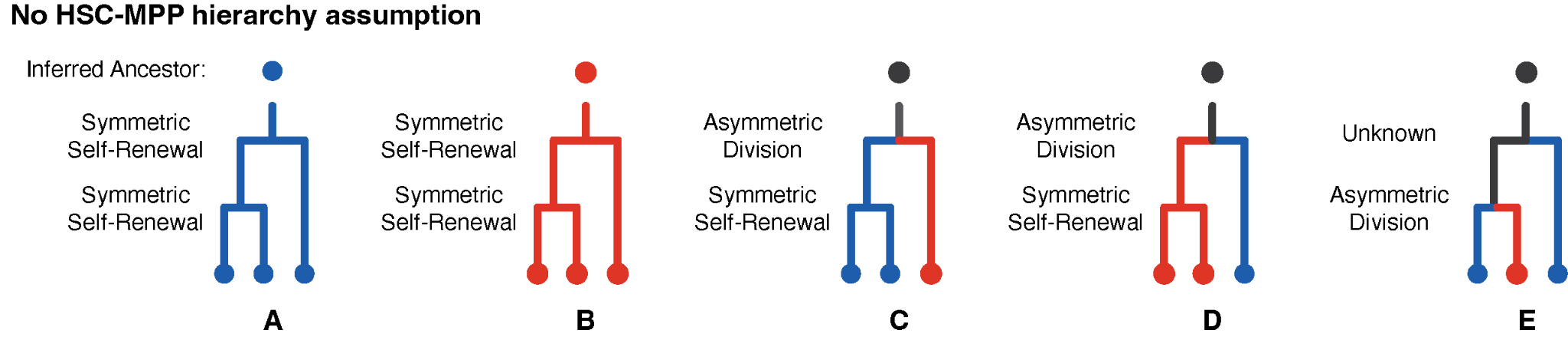


Supplementary Figure S2: Ancestral cell identity inference with no assumption of HSC or MPP hierarchy.

***Supplementary Figure S2: Ancestral cell identity inference with no assumption of HSC or MPP hierarchy.*** *Five scenarios of differing tip states are shown; the tip state represents the observed cell identity.*

*HSC identity is shown as blue, MPP identity is shown as red.*

In our phylogenies, coalescences represent cell divisions of ancestral cells whose progeny have been captured as observable cells (tips on the tree). Comparison of the observed cell identity between closely related tips allows inferences of the identity of their most recent common ancestor (MRCA) and the nature of the ancestral cell division captured as a coalescence on the phylogenetic tree.

As an illustrative example above, if two closely related observed (‘tip’) cells are HSCs (scenarios A and C), then it is inferred that their most recent common ancestor was also an HSC. This HSC must have symmetrically divided to create two daughter HSCs, with both lines of descent also generating HSCs that were eventually sampled as the observed cells. From this inferred cell identity of their most recent common ancestor, if the cell state of the next closest relative is also an HSC, then their most recent common ancestor is similarly inferred to be an HSC (scenario A). HSCs coalescences are in blue, while MPPs are in red (scenarios B and D). Neighbouring tip states that differ in cell type (*e.g.,* 1 HSC and 1 MPP as in scenario E) can arise in two ways. First, there may have been an ancestral asymmetrical cell division generating one HSC and one MPP initially, with subsequent progeny along both lines of descent retaining these identities until sampling. Alternatively, the same tip states could also occur via a symmetrical self-renewing division of either MPP or HSC, followed by a later cell type change (*e.g.,* via asymmetric cell division or direct change) of one of the daughter cells. Either way, one cell type change from HSC to MPP (or MPP to HSC) is required to explain these tip states; therefore, we mark their ancestral coalescence as blue/red. In these scenarios, because we cannot infer the cell identity of the MRCA, the upstream lineage is subsequently labelled in black. These principles can be applied to all coalescences in the observed phylogenetic trees (Fig.2a-b, ED Fig.2, scenarios A-E above). This intuition does not rely on any assumptions of ontogeny, such as the hierarchy of HSCs over MPPs.


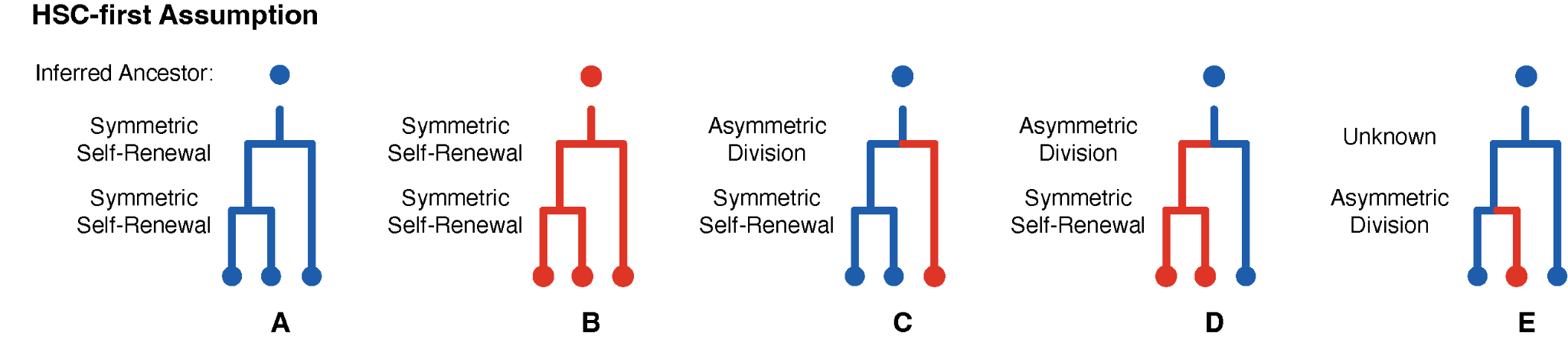


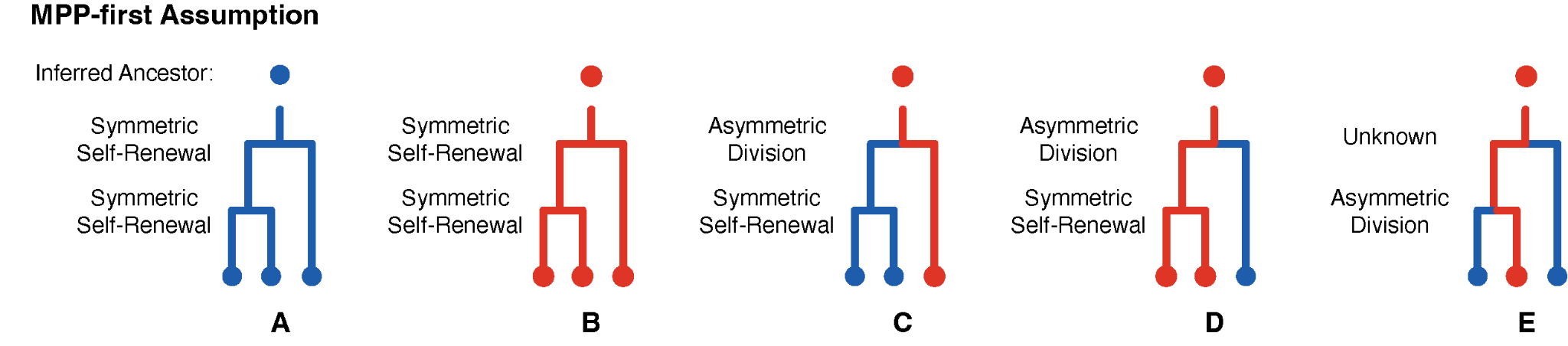


Supplementary Figure S3: Ancestral cell identity inference assuming an HSC common ancestor or MPP common ancestor

***Supplementary Figure S3: Ancestral cell identity inference assuming an HSC common ancestor (top) or MPP common ancestor (bottom).*** *Scenario and cell identity colouring is as described in Supplementary Figure S2.*

However, current models of the hematopoietic hierarchy dictate that HSCs give rise to MPPs (HSC>MPP). With this assumption made (top row “HSC-first assumption), one can label more of the branches and coalescences assigned as ‘black’ in the logic detailed above. For example, assuming an ‘HSC-first’ hierarchy, the common ancestor for scenarios C-E is now inferred to be an HSC, and the unobservable ancestral division in scenario E is inferred to be an HSC self-renewal. In the ‘MPP-first’ assumption (lower row), the inverse is inferred. These heuristics are applied to all coalescences in the observed phylogenetic trees.

We then asked how many cell state transitions are required to explain the tip states given an HSC-first or an MPP-first model. To perform this comparison, for each tree, we subsampled the largest category of HSC and MPPs so that there were equal numbers of MPP and HSC tips. To reduce the risk of the downsampling being unrepresentative the subsampling was conducted 10,000 times for each tree, and the average number of required transitions under the two unidirectional models was calculated. We then counted the total number of transitions required to result in the observed cell type tips. It was observed that the number of transitions required was similar for HSC-first and MPP-first and that there was no consistent pattern of one being higher than the other (Fig.2d). It was then natural to ask whether our cell type information was at all informative and so we randomly permuted the tip cell types and then resolved the tree in an HSC-first fashion. This sub-sampling and permutation was carried out 10,000 times and, as expected, the number of changes required by either the MPP-first or HSC-first models were generally far fewer than is consistent with the null model that all balanced cell type categorisations require the same number of tree-based transitions to explain the tip phenotype under a unidirectional model. In summary, both HSC-first and MPP-first models are less parsimonious, i.e., requiring more cell state changes, than the model first presented in which no assumptions are made about a hierarchy between HSCs and MPPs. The most parsimonious model would be that of HSC and MPP lineages being derived in parallel during similar development periods from non-overlapping common ancestors.

**A simple 3 state model for Murine Progenitor Ontogeny**

To formalise the above ideas in the context of a simple model of HSC and MPP ontogeny, we considered the state of all cells prior to 10 mutations in molecular time as being in an embryonic precursor state (EMB), given that haematopoietic and colonic lineages remain uncommitted until at least this time (ED Fig.3). We then assumed that in each unit molecular time there is a fixed probability of transitioning out of this embryonic state into either an HSC state, $p_{EMB->HSC}$, or an MPP state, $p_{EMB->MPP}$. Furthermore, there is a fixed probability of transitioning from an HSC to an MPP, $p_{HSC->MPP}$, and from an MPP to an HSC, $p_{MPP->HSC}$. Thus, the evolution of the cells down the tree is governed by a discrete time Markov chain process. The likelihood of the observed tip cell types is calculated using a hidden Markov tree approach (Methods). Maximum likelihood estimates of the model parameters are obtained by maximising the sum of the log-likelihoods across mouse-specific phylogenetic trees. Finally, for each mouse, the most likely sequence of unobserved states for the nodes of the phylogenetic tree is calculated using the fitted model parameters.

We performed the maximum likelihood estimation using the R package “bbmle”. The maximisation was performed on logit transformed quantities: $p_{HSC->MPP}$, $p_{MPP->HSC}$, $\frac{p_{EMB->HSC}}{p_{EMB->HSC}+p_{EMB->MPP}}$and $p_{EMB->HSC}+p_{EMB->MPP}$. Whilst we were able to obtain parameter estimates and Hessian-based standard errors, the profile-based estimation of confidence intervals did not work in all cases. So, to obtain more robust estimates in the CIs of the model we implemented a Stan-based Bayesian version of the model using the directly calculated likelihood as described above. Uniform priors on the unit interval were assumed for $\frac{p_{EMB->HSC}}{p_{EMB->HSC}+p_{EMB->MPP}}$ and $p_{EMB->HSC}+p_{EMB->MPP}$ and uniform priors on the interval (0-0.5) were assumed for both $p_{HSC->MPP}$ and $p_{MPP->HSC}$ . The model was run with four chains, each for 10,000 iterations.

**Separate young and old mouse cohorts provide optimal model fit**

We compared fitting the model with a per-mouse, per-age, and pan cohort strata. A likelihood ratio test analysis revealed that the best model is an age-specific model where parameters are estimated separately in old and young mice (Supplementary Table S1).

| Model | Degrees of freedom | Log Likelihood | AIC | Likelihood Ratio Test |
| --- | --- | --- | --- | --- |
| Pan Cohort | 4 | -840.2 | 1,688.4 |  |
| Age-Specific | 8 | -800.2 | 1,616.5 | vs. Pan Cohort: P=1.78e-16 |
| Mouse-Specific | 24 | -796.2 | 1,640.3 | vs. Age Specific: P=0.945 |

Supplementary Table S1: Log likelihood values and Akaike information Criterion (AIC) assessing model fit

***Supplementary Table S1.*** *Log likelihood values and Akaike information Criterion (AIC) assessing model fit.* *A lower AIC value indicates a better model fit.*

**Young and old mice exhibit differing patterns of differentiation**

Applying our hidden Markov tree approach, we fitted an HSC-first model where,

$\frac{p_{EMB->HSC}}{p_{EMB->HSC}+p_{EMB->MPP}}$

is fixed at unity. That is, all EMB must transition to an HSC before any emergence of MPPs (HSC-first). In the context of this simple model, we can reject the HSC-first model across the combined age group model (p=1.11e-18) and also for the old group (p=1.38e-19). However, we were unable to reject the HSC-first model for the young animal group (p=0.397).

Examining the types of cell-state transitions in the trees, we observed that aged animals exhibit several independent transitions from the embryonic precursor state followed by relatively few transitions between HSC to MPP or vice versa (Supplementary Fig.S4). In contrast, young animals exhibit a tendency towards HSC-first followed by a relative abundance of HSC->MPP transitions (Supplementary Fig.S4). Both HSC-specification and MPP-specification occur within the first 50 mutations molecular time (Supplementary Fig.S5). The cell identity transition rates, per unit molecular time, are listed below (Supplementary Table S2) and were used to generate Fig.2e.

|  | $p_{EMB->HSC}$ | $p_{EMB->MPP}$ | $p_{HSC->MPP}$ | $p_{MPP->HSC}$ |
| --- | --- | --- | --- | --- |
| Young Donors | 0.158 | 0.037 | 0.0164 | 0.0070 |
| Aged Donors | 0.036 | 0.034 | 0.0013 | 0.0006 |

Supplementary Table S2: Cell identity transition rates per unit molecular time derived using the hidden Markov approach.

***Supplementary Table S2.*** *Cell identity transition rates per unit molecular time derived using the hidden Markov approach.*

The above result are fairly consistent with the Stan based results (Supplementary Table S3) for which we show the medians of the marginal posterior distribution followed by the 95% credibility intervals:

|  | $p_{EMB->HSC}$ | $p_{EMB->MPP}$ | $p_{HSC->MPP}$ | $p_{MPP->HSC}$ |
| --- | --- | --- | --- | --- |
| Young | 0.43(0.11 - 0.9) | 0.063(0.0048 - 0.27) | 0.017(0.014 - 0.022) | 0.0078(0.0029 - 0.016) |
| Aged | 0.04(0.025 - 0.068) | 0.038(0.025 - 0.064) | 0.0014(0.00079 - 0.0022) | 0.00071(0.00022 - 0.0015) |

Supplementary Table S3: Cell identity transition rates per unit molecular time derived using the Stan-based Bayesian model.

***Supplementary Table S3.*** *Cell identity transition rates per unit molecular time derived using the Stan-based Bayesian model. 95% confidence intervals are shown in parentheses.*

Of note, the mode of the marginal posterior distribution of $p_{EMB->HSC}$ peaks at 0.19, which is reassuringly close to the maximum likelihood estimate of 0.158.


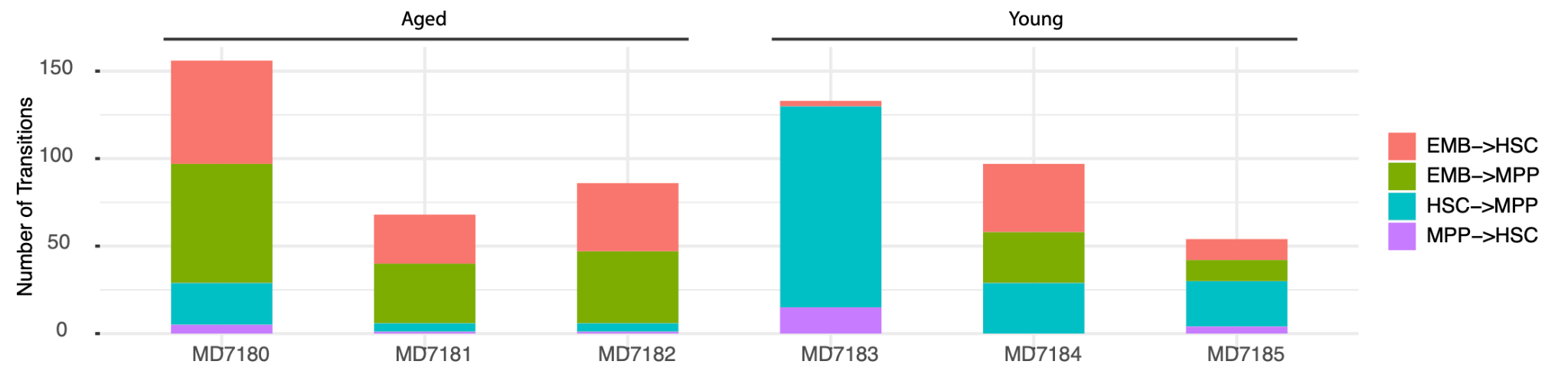


Supplementary Figure S4: Transition Type Counts

***Supplemental Figure S4: Transition Type Counts.*** *The old mice exhibit an abundance of approximately equally prevalent EMB->HSC and EMB->MPP transitions followed by relatively few transitions to the eventual observed cell types. The young mice exhibit relatively fewer EMB->HSC and EMB->MPP and then a relative abundance of HSC->MPP transitions.*


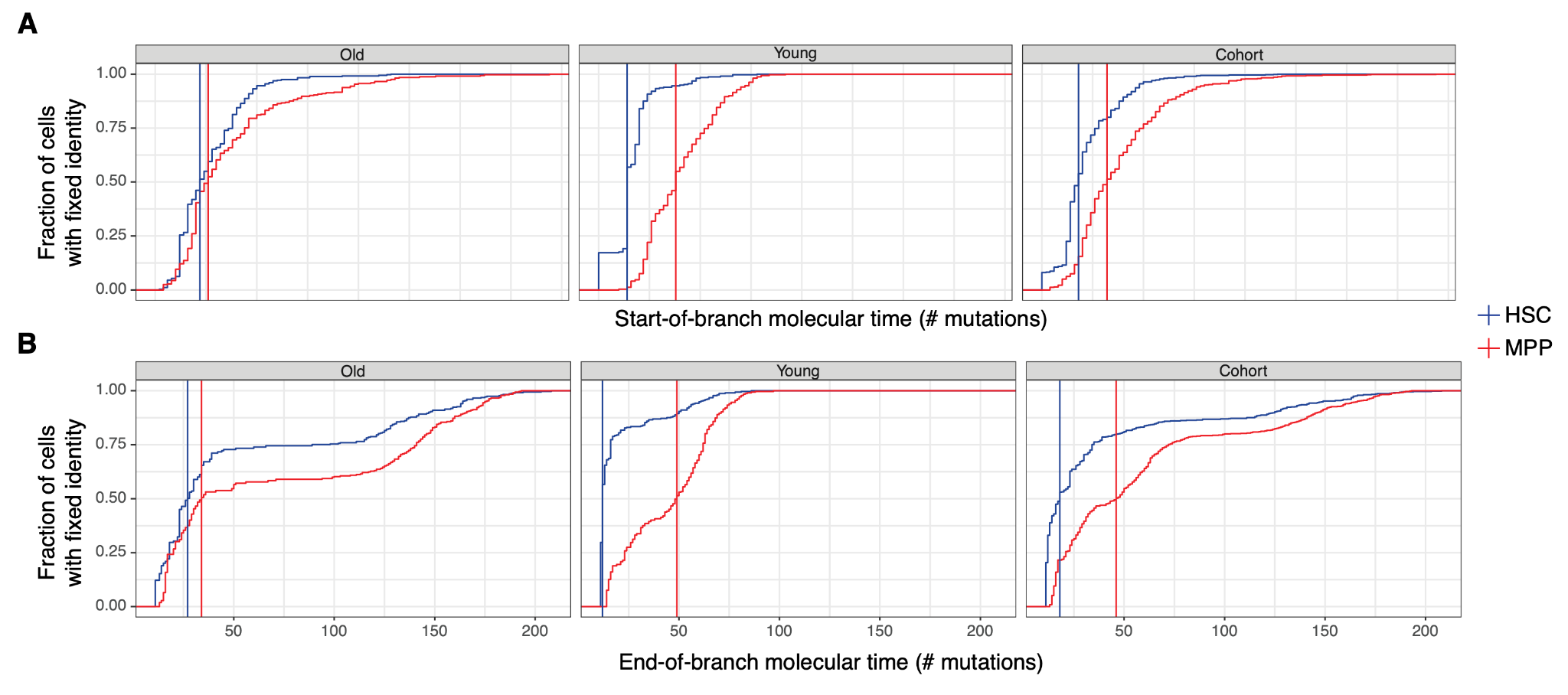


Supplementary Figure S5: Cumulative Distribution of Specification Timing

***Supplementary Figure S5: Cumulative Distribution of Specification Timing.*** *For each colony we use the molecular time of the* ***A)*** *start of the branch, or* ***B)*** *end of the branch on which the ancestral lineage first transitions to the observed cell type as an upper bound for the timing of its transition to its final observed state. The panels show the cumulative distribution of these upper bounds calculated form the most likely sequence of transitions inferred using the age-specific model and the pan cohort model. Vertical lines indicate the time at which 50% of the sampled cells have specified identity.*

# Supplementary Note 3: Quality control of targeted duplex-sequencing

We expected that somatic clones in mice might be rare events at small clone sizes, thus would require a sensitive detection assay. High-depth sequencing can be used for detection of subclonal variants, but with increasing coverage, the error-rate intrinsic to short-read sequencing can obscure true low variant allele fraction (VAF) variants. To circumvent this sensitivity limit, read-level error-correction approaches are necessary. Thus, we applied duplex-consensus sequencing, which offers among the highest sensitivity for subclonal variant detection. In duplex-sequencing, each initial dsDNA molecule is uniquely barcoded such that reads derived from complementary 5’ and 3’ strands are linked, but also distinguishable. Detected variants must be present on both uniquely barcoded strands of the initial dsDNA fragment to pass bioinformatic filtration (Supplementary Fig.S6). By enforcing that variants are present in reads derived from both of the matched complementary strands of DNA, one can eliminate the majority of sequencer-induced artefacts that usually hamper sensitivity. To apply this technology to murine clonal haematopoiesis (CH), we developed a target panel of the mouse homologs of genes most frequently mutated in human CH (Methods, Supplemental File 2).


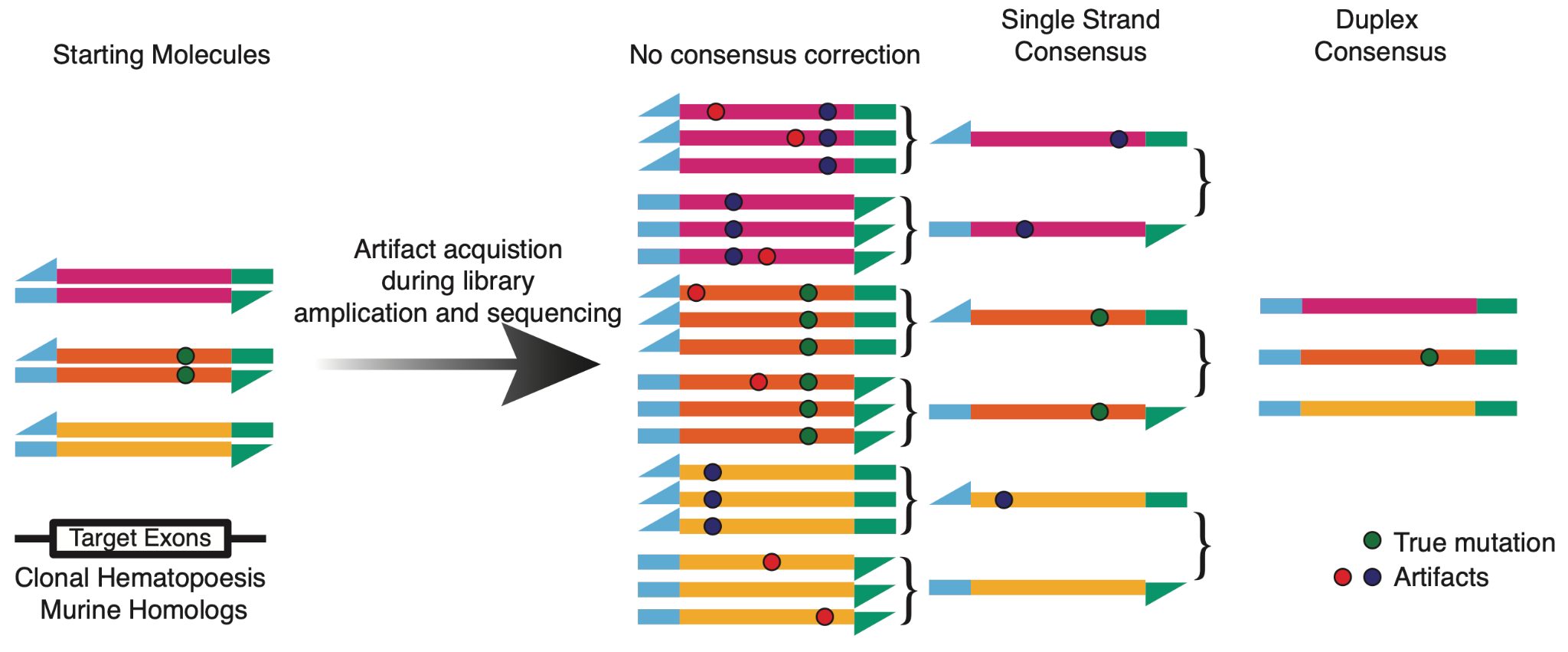


Supplementary Figure S6: Supplementary Figure S6: Error correction strategy in targeted duplex sequencing.

***Supplementary Figure S6: Error correction strategy in targeted duplex sequencing.*** *PCR during library preparation and sequencing introduce low-frequency artefacts. Duplex barcodes allow grouping of PCR duplex reads from a single DNA library molecule (read families) and single-strand read consensus generation. Next, single strand consensus reads from complementary strands on initial dsDNA are matched to generate a duplex consensus. To build a duplex-consensus read, we required at least 3 reads in each supporting read family (i.e., at least 3 sequenced PCR duplicates of matched top and bottom strands from an original dsDNA molecule).*

*Coverage requirements to generate a duplex consensus***:** To generate a duplex-consensus read, an initial DNA molecule must be sequenced multiple times with reads from matched 5’ and 3’ strands sufficiently represented. To ensure that clone detection sensitivity would not be limited by input genomic DNA (*i.e.*, the libraries contained sufficient genomic complexity), we input at least 100,000 genomic equivalents (or at least 1650 ng of genomic DNA) into our library preparations. High library complexity decreases the probability of matched 5’ and 3’ reads being sequenced by chance; thus, even with a target panel enrichment, extremely high sequencing depth is required to capture library complexity in duplex consensus reads. Median raw, non-deduplicated coverage spanned 1,000,000X to 3,000,000X at targeted loci per sample. This correlated with a single-strand consensus coverage spanning 60,000X-120,000X, which, after 5’ and 3’ linkage, further collapsed to duplex consensus coverage spanning 30,000X-40,000X (Supplementary Fig.S7). Duplex coverage at specific exons within targeted genes was variable between samples (Supplementary Fig.S8)


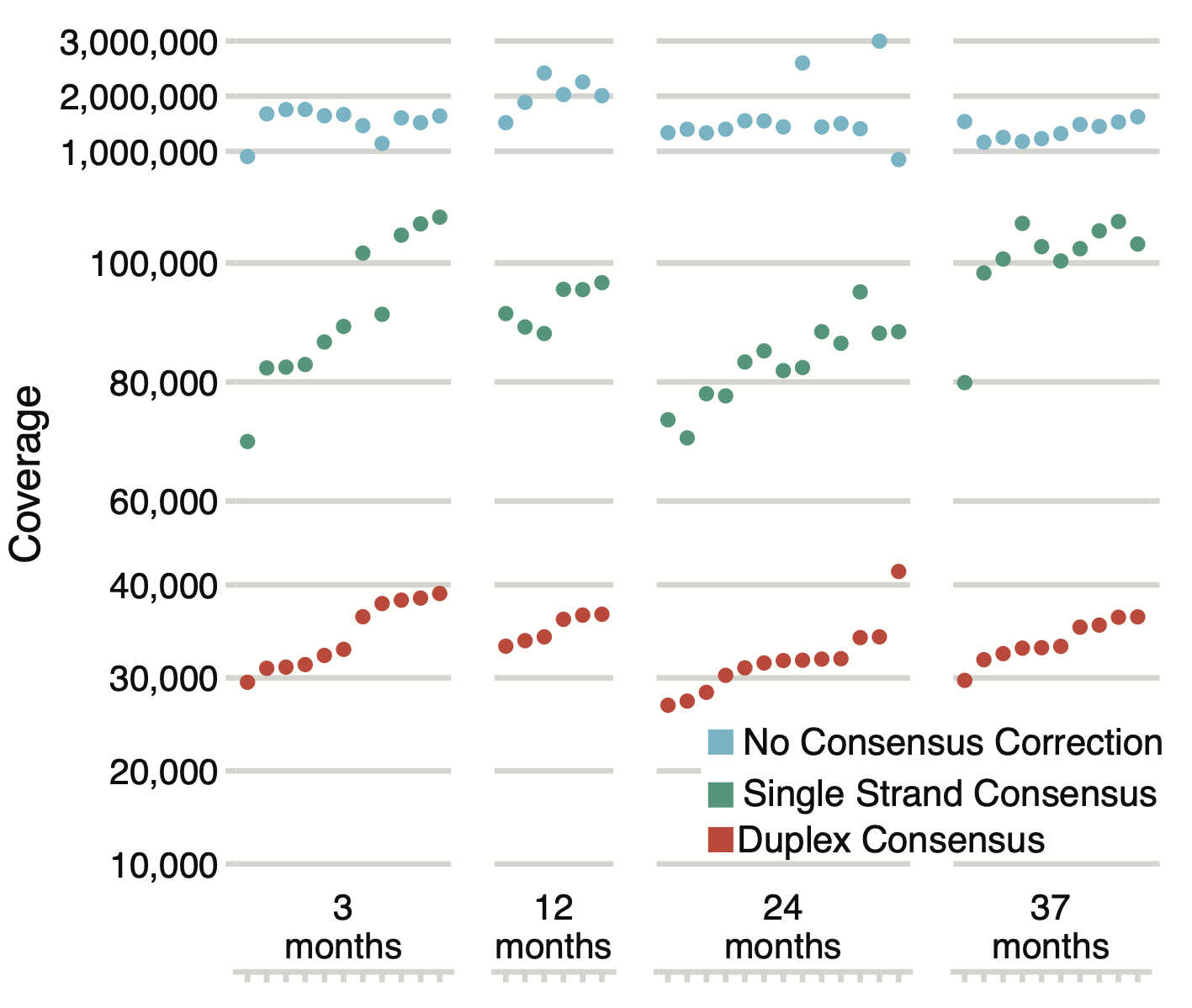


Supplementary Figure S7: Sequencing coverage at targeted loci.

***Supplementary Figure S7:*** *Sequencing coverage at targeted loci for all samples in Fig.4A. The relationship between raw (not deduplicated), single strand consensus, and duplex consensus coverage is shown.*


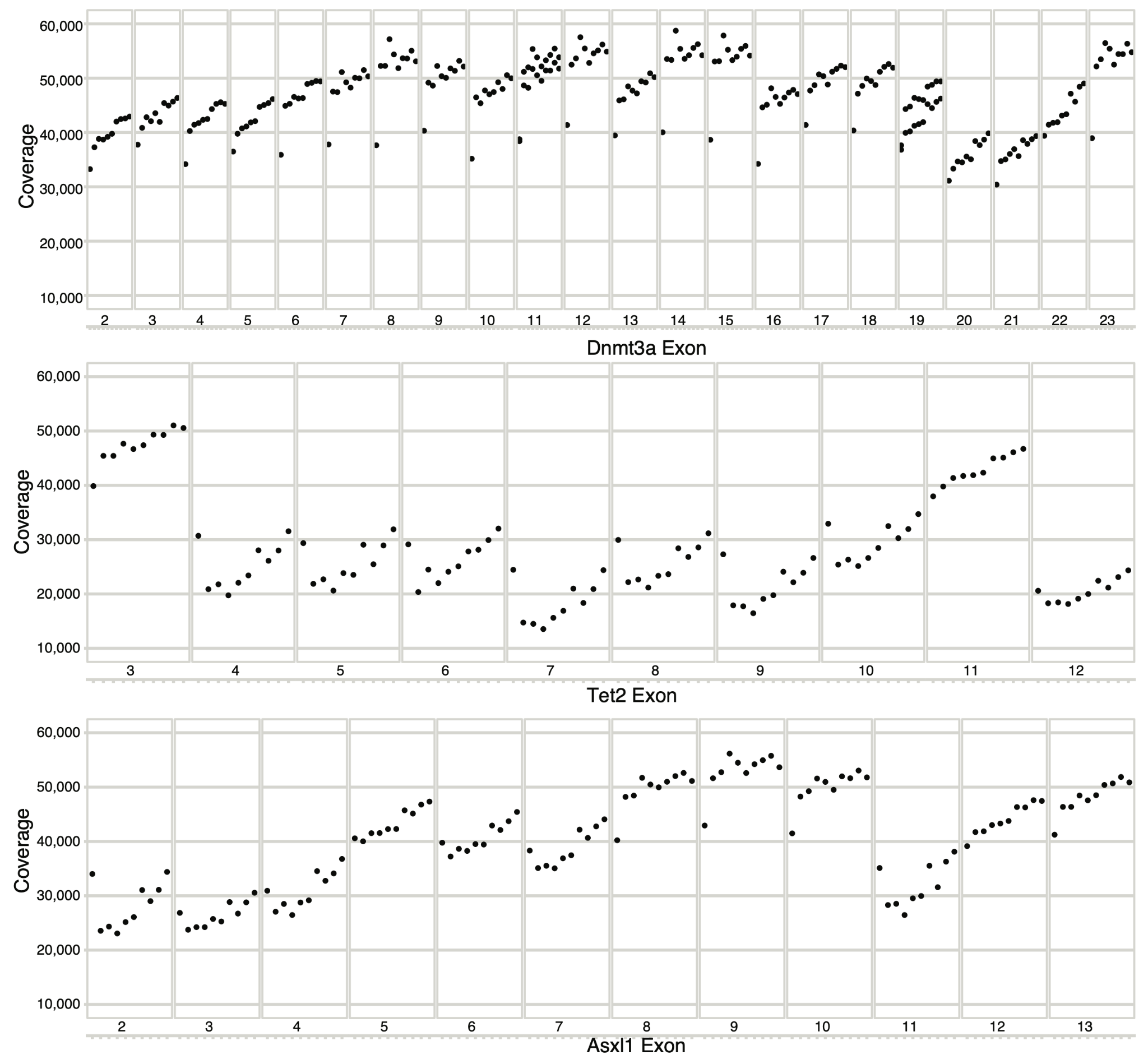


Supplementary Figure S8: Duplex coverage at coding exons in Dnmt3a, Tet2, and Asxl1

***Supplementary Figure S8:*** *Duplex coverage at coding exons in Dnmt3a, Tet2, and Asxl1 for a series of aged samples.*

*Mutation filtering:* To supplement the sensitivity afforded by duplex sequencing, stringent read- and variant-level filters were applied to reduce the presence of false positive mutations or spurious calls. Without filtration, we observed an enrichment of C>A mutations (Supplementary Fig.S9), reminiscent of mutation signature SBS45,which is likely attributable to oxidative damage during sequencing^66,67^. Such oxidative damage mutations likely arose after duplex barcode attachment, were enriched at read ends, and likely caused mutations within the duplex barcode sequence. Due to mutations in duplex barcodes, a read family derived from a single initial dsDNA molecule (a singleton) would erroneously appear as derived from an additional read family (a doublet). This observation led us to apply a stringent series of filters (Methods), after which the trinucleotide spectra of variants detected in duplex sequencing more resembled that seen with blood (Supplementary Fig.S9).


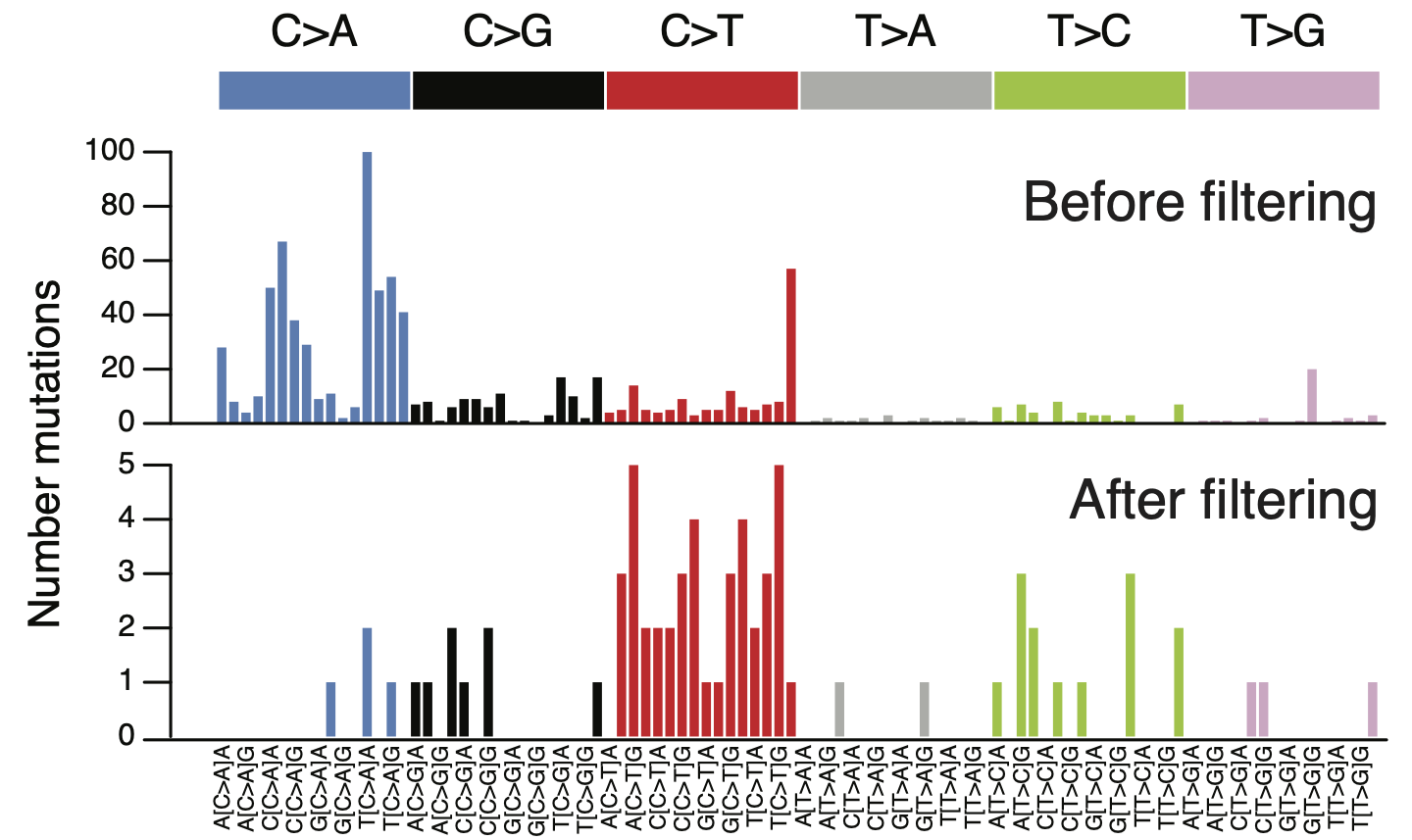


Supplementary Figure S9: Trinucleotide spectra of duplex-sequencing variants before and after post-processing filters.

***Supplementary Figure S9:*** *Trinucleotide spectra of duplex-sequencing variants before and after post-processing filters. See Methods for filtering strategy details.*

*Clone size calculation:* Given the differences in coverage between loci, we normalised the variant read counts to allow accurate clone size comparisons between samples. In general, clone size for a given variant is defined as:

$$Clone size = \frac{Mutant allele read count}{Total read count}$$

For very small clones, there is a degree of stochasticity affecting if sufficient mutant read alleles will be converted to duplex consensus reads to allow detection. Duplex clones supported by very few mutant allele reads would have a low numerator, thus clone size estimations may be skewed. Given more single-strand consensus reads are generated than duplex consensus reads (Supplementary Fig.S7), we reasoned that mutant allele reads would be relatively more abundant within single-strand consensus reads – that is, mutant allele reads would be present among the reads ‘discarded’ due to insufficient evidence to generate a duplex consensus. To normalise clone size, especially in low-magnitude clones, we used de-duplicated single-strand consensus reads, as follows:

$${Clone size}_{corrected}=\frac{{Mutant allele read count}_{single strand consensus}}{{Total read count}_{single strand consensus}}{}$$

By using the de-duplicated single-strand consensus reads for clone size calculation, the numerator (variant allele count) and denominator (coverage) both increase, reducing any skewing that may be present in clone size calculations from duplex consensus reads. All clone sizes depicted on dot plots are calculated in this manner.

*Biological replicates:* To validate reproducibility within the targeted duplex-sequencing library preparation and variant calling pipelines, we assessed clone prevalence in biological replicate samples. For each replicate, peripheral blood was separately collected (in different tubes) and underwent genomic DNA extraction independently. Thus, the genomic DNA “pools”, while derived from the same sample mouse, were purified in separate reactions. Replicate DNA samples underwent duplex library preparation and variant calling as described in Methods. As shown in Supplementary Figure S10, clone detection is concordant between paired replicates. Clones unique to a single replicate were at the limit of detection for the specific locus, and thus it is likely in the paired replicate that insufficient variant reads were sequenced to generate duplex consensus read support. Such borderline detectable clones will likely be detectable within single-strand consensus reads, which carry nearly double greater read depth, though at the expense of duplex sensitivity. We examined single-strand consensus reads from the biological duplicate samples and were able to “rescue” missing variants from the paired replicate sample, in about half of cases (Supplementary Figure S10). This confirms that much of the missing replicate clones were lost during duplex consensus building, for example when a clone has insufficient top or bottom strand support to create a duplex read.


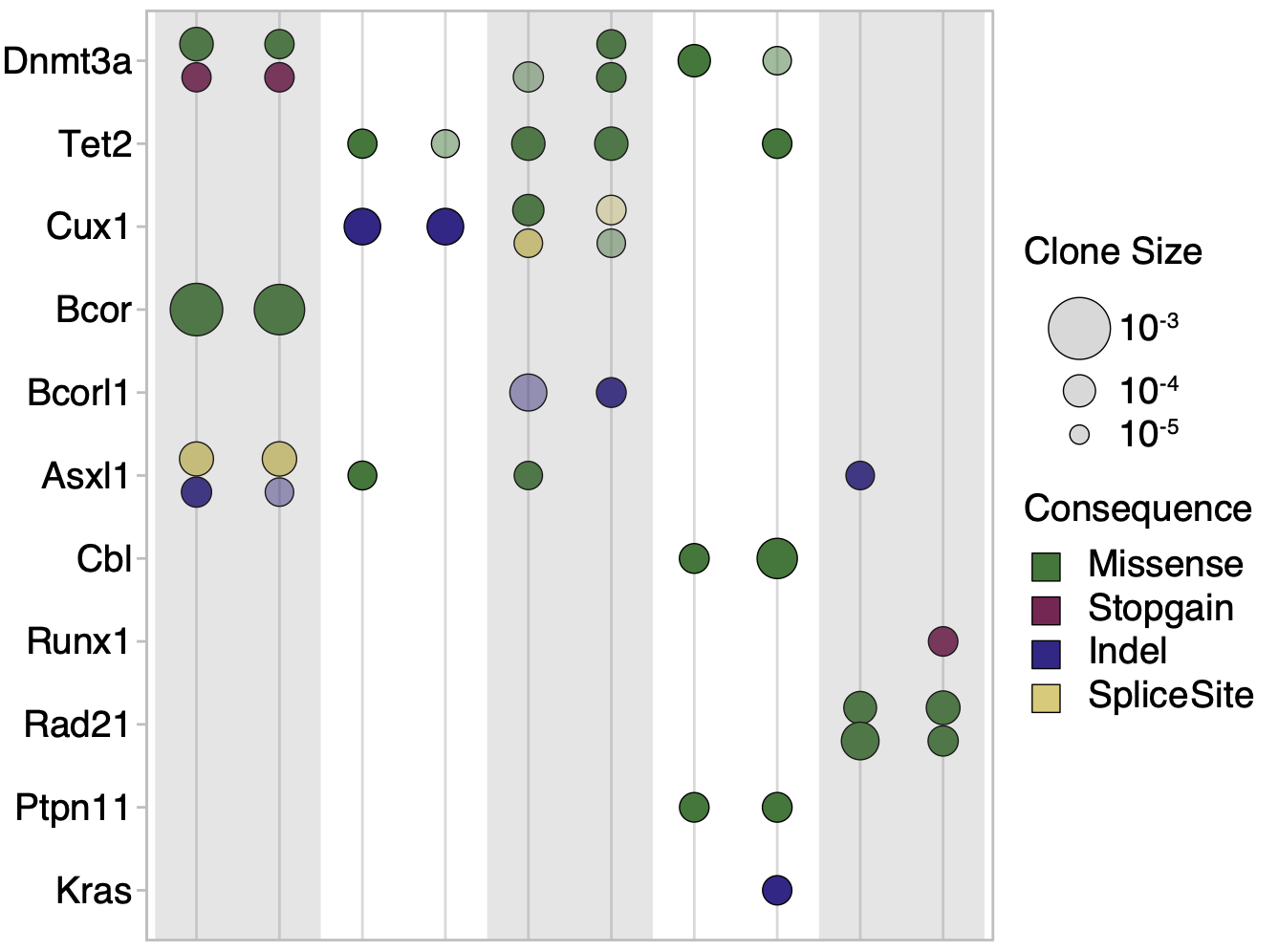


Supplementary Figure S10. Native CH in biological replicate samples

***Supplementary Figure S10. Native CH in biological replicate samples.*** *Shaded and unshaded pairs represent duplex libraries separately prepared from an identical initial blood sample. Clones are presented as described in Fig.4a. Transparency indicates a clone that was only detectable within single strand consensus reads but not duplex consensus reads.*

*In silico estimation of the sensitivity and specificity of duplex-sequencing results:* We next sought to understand if the degree of clone concordance between biological replicate samples was consistent with the sensitivity of our assay. We consider a simple model for SNVs of conditional base calling probabilities for the reference base (R), a mutant base (A) and the two other bases (B,C). For an individual read (or read family/bundle) the probability of observing the bases is modeled in the following manner:

$$P(Base is A)=$$

$P(DNA Molecule is mutant A at site) *$
 $P(Base called as mutant A|DNA Molecule is mutant A at site)$

$$+P(DNA Molecule is not mutant at site)*$$

$P(Base called as mutant A|DNA Molecule is not mutant at site)$.

Now $P\left( DNA Molecule is mutant at site \right)=\frac{Aberrant Cell Fraction}{ploidy}=VAF$ where for economy we now use the term (true) VAF to characterise the clone. Moreover, we assume there is a base calling error rate (“epsilon”) $\epsilon$. It is assumed that this results in the one of the 3 incorrect bases to be called with equal probability of $\epsilon/3$:

$$P\left( Base is Reference \right)=\left( 1-VAF \right)\left( 1-\epsilon\right)+VAF\frac{\epsilon}{3}$$

$$P\left( Base is A \right)=VAF\left( 1-\epsilon\right)+\left( 1-VAF \right)\frac{\epsilon}{3}$$

$$P\left( Base is B \right)=VAF\frac{\epsilon}{3}+\left( 1-VAF \right)\frac{\epsilon}{3}=\frac{\epsilon}{3}$$

$$P\left( Base is C \right)=\frac{\epsilon}{3}$$

For a given bait set wide depth of sequencing, $depth$, a given site has depth that is Poisson distributed with mean $depth$. For a clone to be detected it is only required that at least 2 mutant reads are observed. We assume we have a known clone, with VAF=1^-4^ or VAF=1^-3^, and with mutant allele A. The A clone is discovered if there are 2 or more mutant “A” reads, and no other mutant reads (“B” or “C”). With these criteria, we can plot the sensitivity for a given error rate, $\epsilon$, shown below in Supplementary Figure S11.


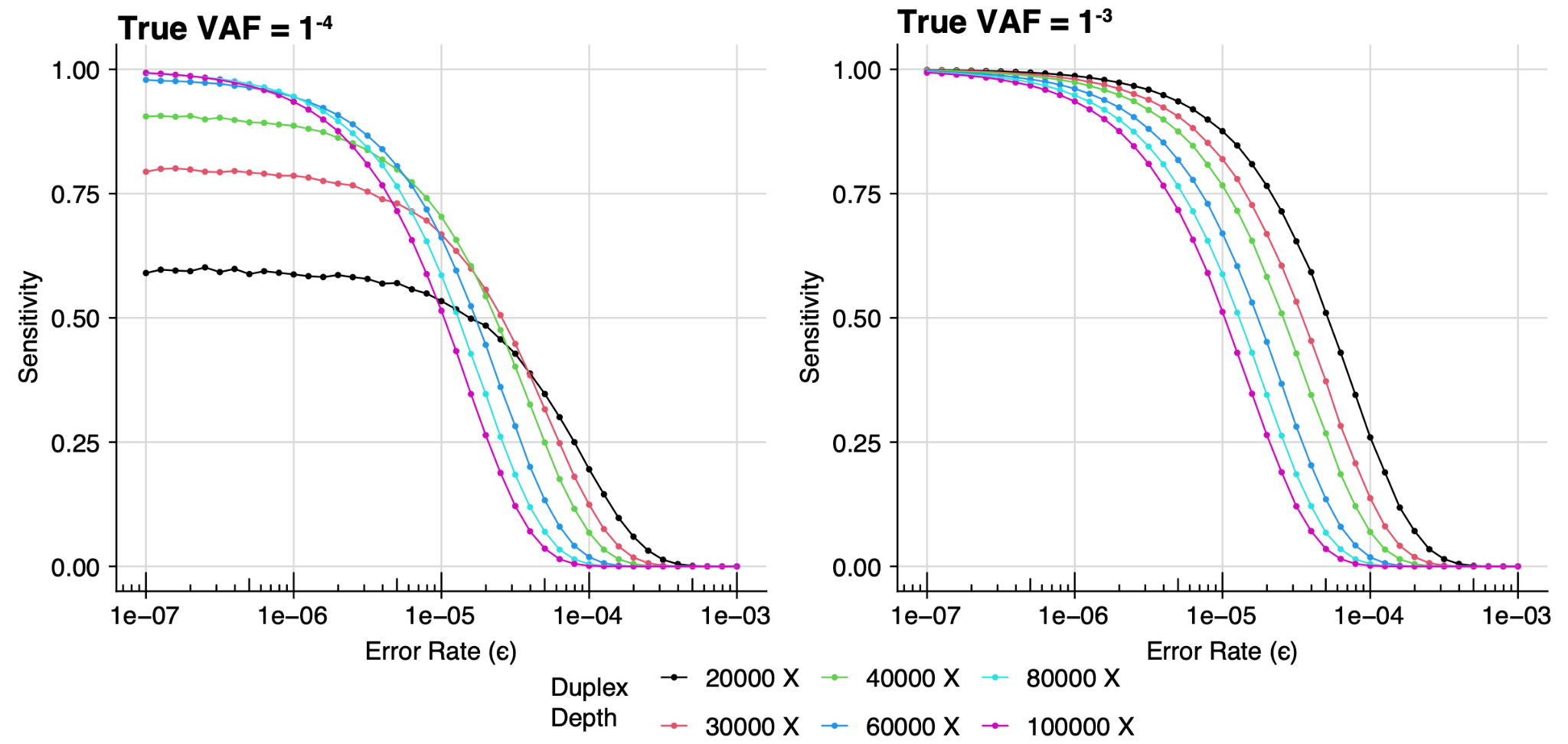


Supplementary Figure S 11: True clone discovery across error rates using multinomial modeling

***Supplementary Figure S11: True clone discovery across error rates using multinomial modeling.*** *Estimated sensitivity of detecting a variant at a given site with true VAF 1^-3^ (left) or 1^-4^ (right) across increasing error rates. A range of duplex depth at variant sites are shown.*

The above plots show that using single strand consensus sequencing with error rate of ~3^-5^ at depth 60,000x provides a sensitivity of 30% for clone sizes of VAF=1^-3^ or less. However, using duplex depth of 30,000x with an error rate of 1^-6^ to 1^-7^ (as described in Kennedy *et al*.^62^) provides a sensitivity of >75%.

If we assume the extreme (and implausible) case of error-free sequencing, then the clone detection sensitivity is purely governed by the binomial distribution with a probability of True VAF. Importantly, even if the sequencing was error-free, we would not expect there to be concordance of clone detectability in different samples. In the Supplementary Figure S12 below we can see that for error-free sequencing at depth 20,000X, we would have a concordance of around 60%. This aligns with the observed duplex clone concordance seen in the biological replicate samples shown in Supplementary Figure S10.


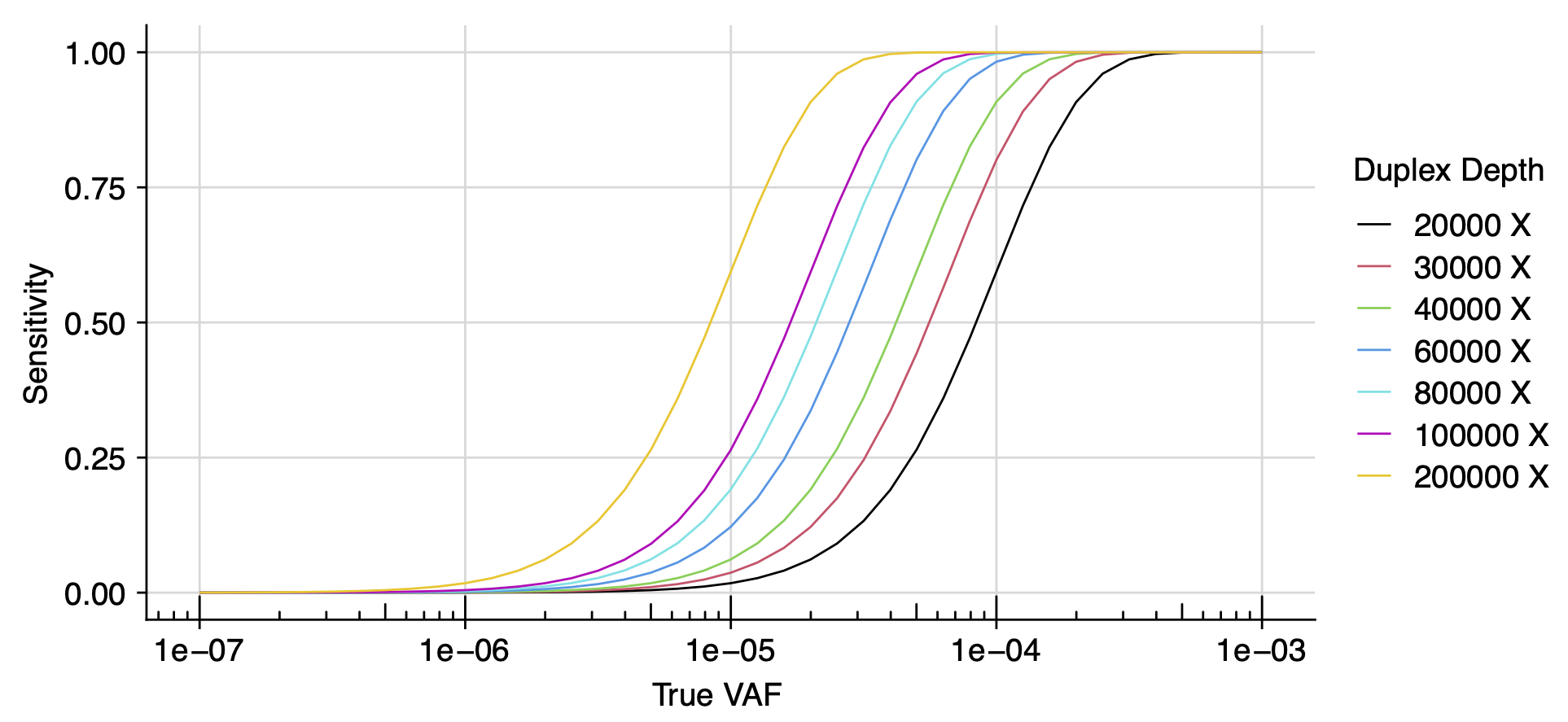


Supplementary Figure S12: True clone discovery with error-free sequencing

***Supplementary Figure S12: True clone discovery with error-free sequencing.*** *The estimated sensitivity for clone detection at increasing VAFs in the scenario of error-free variant detection. In the absence of an error rate, detection sensitivity can be described with a binomial distribution. A range of duplex depth at variant sites are shown.*

Finally, we can estimate the probability of false positive clone detection at a given error rate $\epsilon$. As shown below, when querying a range of feasible duplex-sequencing sensitivities and duplex-corrected sequencing depths, a false positive clone is far less likely than a false negative clone (missing a true event). As an illustrative example, for duplex depths 20,000X to 30,000X and the duplex error rate of <8e-04 (estimated error rate of <1e-06), the false positive rate is <0.01. (Supplementary Figure S13).


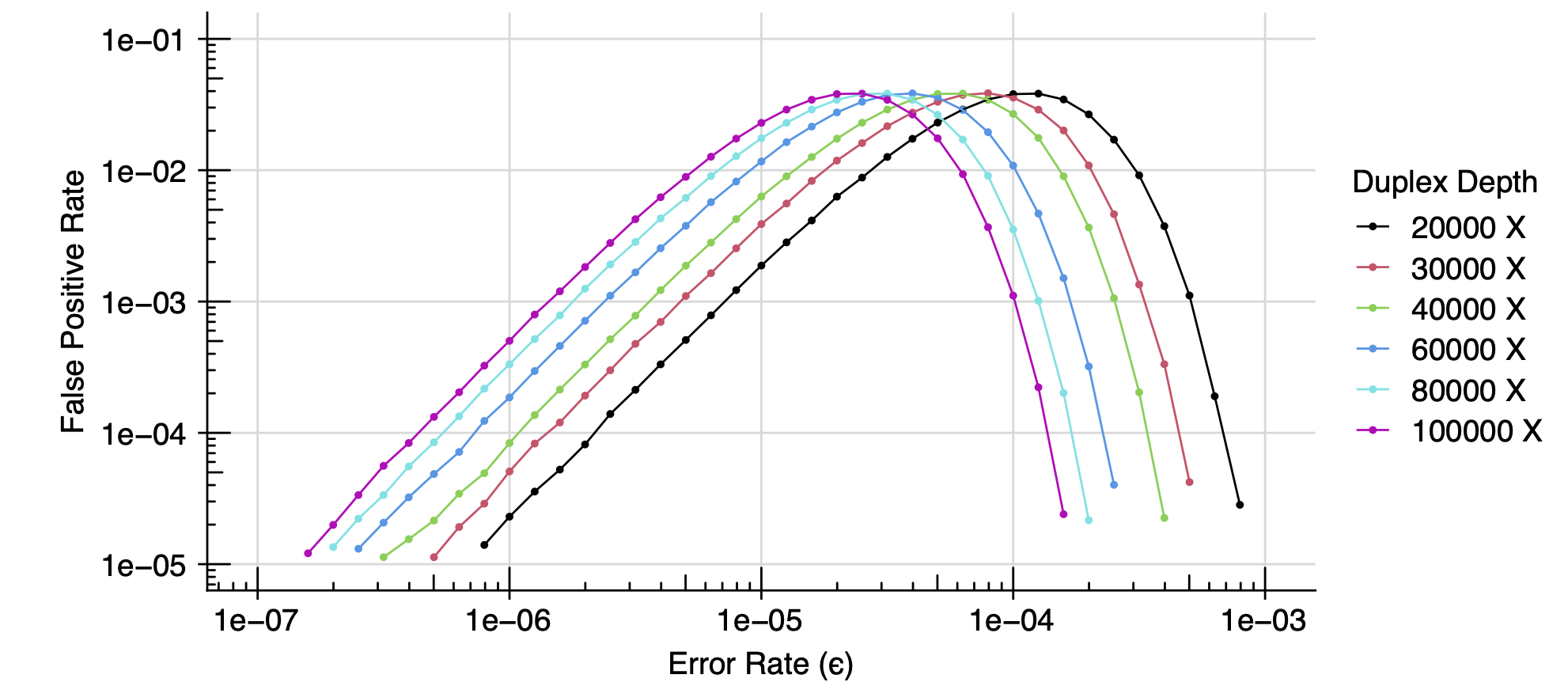


Supplementary Figure S13: False positive variant detection

***Supplementary Figure S13: False positive variant detection.*** *The estimated incidence of incorrectly detecting a variant at a given site is shown, using multinomial modeling of detection error rate and site-specific duplex depth.*

*Concordance of duplex sequencing data explored through mixing mutant and wildtype reads:* The in-silico analyses described above suggest that a true variant clone may not be observed due to insufficient duplex read support, and sensitivity increases with additional duplex depth. In this case, an *expected* variant would likely be detectable in single-strand consensus reads (Supplementary Figure S6), which require reduced read support to build a consensus read, and harbour far higher coverage (Supplementary Figure S7), though at the expense of sensitivity.

We performed a mixing analysis using our duplex data, with the aim to evaluate 1) the concordance of calling serially lower VAF clones in different samples, and 2) the degree missing-but-expected clones can be found in single-strand consensus data.

We selected clones with a large detectable clone size, then generated serial dilutions of input mutant file reads with wild-type file reads to simulate diminishing read support and the subsequent detection of an expected variant in duplex reads. Mutant file reads were diluted by the following percentages: 50%, 20%, 10%, 5%, 2%. Five replicates of each random subsample dilution were used as technical replicates. Read dilution was done with raw, unmodified reads; that is, before any mapping or consensus building steps. Mutant reads were mixed with wildtype reads to the same overall read count as the original data, then analysed using the duplex consensus building and variant calling pipeline described herein. In cases where the expected variant was not detected in duplex consensus reads (either due to lack of read support, or failing to pass stringent filters), we examined matched single strand consensus reads for the variant, and often were able to detect the expected clone.

As shown below in Supplementary Figure S14, we observe concordance among technical replicates when the mutant clone is relatively less diluted from the original data, with reduced variant detection in duplex reads as the mutant read support is diminished. The missing variants can be rescued when examining single-strand consensus data. With increasing dilution, the variant eventually lacks sufficient read support to build both duplex or single-strand consensus reads, and is not detectable.


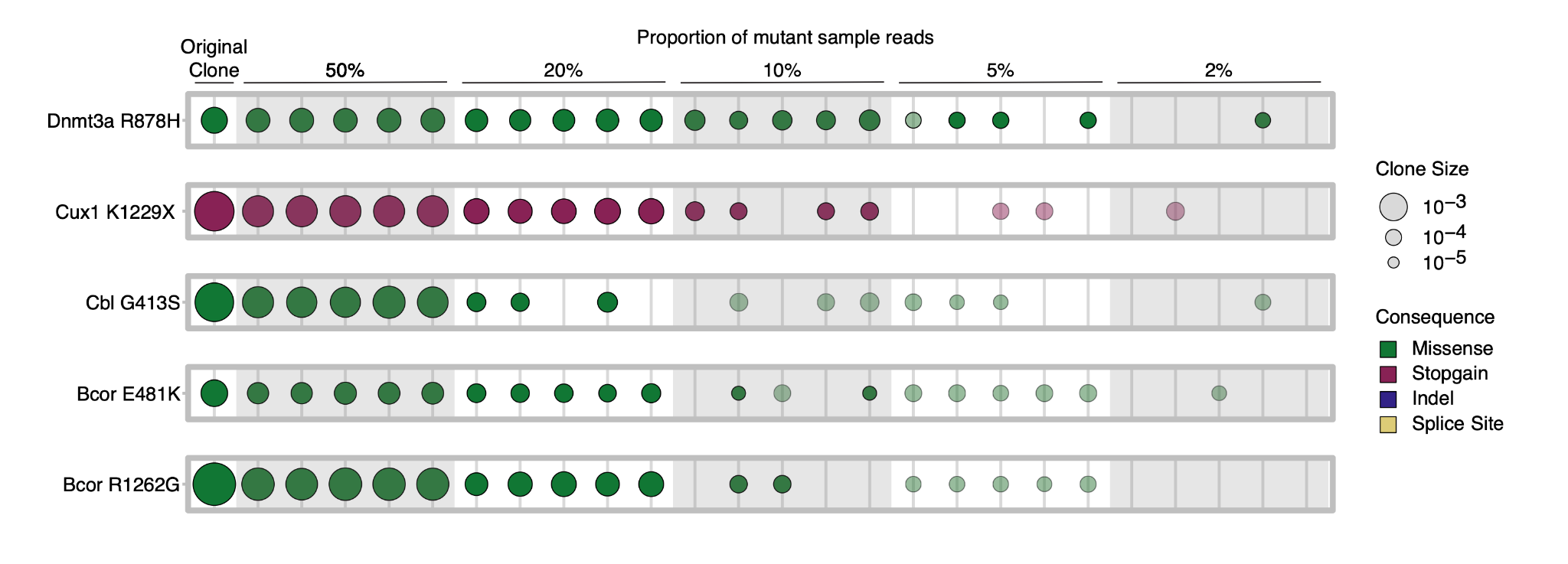


Supplementary Figure S14: Dilution of mutant reads and subsequent variant call concordance.

***Supplementary Figure S14: Dilution of mutant reads and subsequent variant call concordance.*** *For five initially large clones, reads from the original input file (ie supporting the observed clone) were diluted at the indicated proportion with wildtype reads. Five replicates for each dilution factor are grouped. The original clone observed in these unmixed data are shown at the far left column. Clones are presented as described in Fig.4a. Transparency indicates a clone that was not detected with standard duplex filtering, but was detectable within single-strand consensus read.*

# Supplementary Note 4. Inferring population size and division rates from cell phylogenies

The aim of this exercise is to understand the apparent identifiability of the model parameters, seen in Bayesian inferences about the dynamics of HSC populations in mice (and other systems with similar population dynamics), when the phylogeny of a sample of descendent cells is the only available data. These Bayesian inferences were performed using ABC (approximate Bayesian computation) methods. Here the term approximate Bayesian computation refers to a class of Monte Carlo methods for generating samples from posterior distribution, which avoid computation of the likelihood function, by relying on simulation of the model. The more descriptive term *likelihood-free* Bayesian computation is also used. These methods include rejection sampling (pioneered by Pritchard *et al.*^76^), and various regression methods (introduced by Beaumont *et al.* ^29^).

The ABC results reported here were obtained by using the *rsimpop* package^21^ to perform simulations of models of cell population dynamics, and then using the *abc* package^77^ to compute (approximate) marginal posterior densities for modal parameters. The *rsimpop* package allows us to specify a wide range of stochastic growth models based on an underlying birth-death process.

In the case of neutral deterministic growth models, we have exact formulas for the likelihood function, where the model parameter is a sequence of *effective population sizes* (or a sequence of *drift intensities*). For these models, efficient Monte Carlo methods^78^ are available for sampling from the exact posterior distribution of the model parameters. In the case of stochastic growth models which can be approximated by a neutral deterministic growth model, we can obtain an approximate formulas for the likelihood function in which sequence of effective population sizes is replaced by a parameter vector which includes birth rates and death rates as model parameters. We will use approximate likelihood functions obtained in this way to address the issue of identifiability of parameters for various models.

**Likelihood functions for neutral models given phylogeny data**

When we have genome sequences from a sample of single cells taken from an individual donor, we can construct a phylogeny for the sample, with the mutations assigned to branches. From this phylogeny, we can obtain an ultrametric tree, in which the relative lengths of the branches can be estimated (taking account of the number of mutations assigned to each branch). We also know the age $t_{S}$ of the donor at the time point at which the sample was taken. (Here age is measured from the moment of conception.)

From the phylogeny on a sample of $n$ cells, together with the estimated absolute branch lengths, we can label the internal nodes (coalescent event) with integers 2, 3, $\ldots$, $n$, where $n$ is the label on the most recent node (closest to the time of sample collection), and where $2$ is the label on the earliest node on the phylogeny (the root node). We let $S$ be the sequence of node heights $\left( S\left( n \right),S\left( n-1 \right),\ldots,S\left( 2 \right) \right)$, where $S\left( r \right)$ is the height (time in days or years, measured backwards from sample collection) of internal node $r$. These node heights are determined by the branch lengths. The same information is contained in the sequence $T$ of inter-coalescent interval durations $\left( T\left( n \right),T\left( n-1 \right),\ldots,T\left( 2 \right) \right)$. The inter-coalescent interval duration $T\left( r \right)$ is the duration (in days or years) of the time interval during which exactly $r$ lines of descent remain.

We begin by allowing the neutral model to take a very general form, which can be viewed as a generalisation of the neutral Moran model^79^. For now, we measure time $t$ forward from conception ($t$ = 0) when the population of cells contains a single founder cell ($N_{0}$ = 1), which is the zygote. This time $t$ coincides with age (measured from conception). The sequence of distinct time points (ages) at which the population size changes, together with the age $t_{C}$ at the time of sample collection, is recorded as $t$ = $\left( t_{1},t_{2},\ldots,t_{C} \right)$, where 0 $<t_{1}$ $<t_{2}$ $<\ldots$ $<t_{C}$. So we have a sequence of $C$-1 population events which occur before sample collection. We assume that at each population event, these changes in population size occur instantaneously, so that we can define a population size $N_{k}$ which persists throughout the time interval $[t_{k},t_{k+1}\rangle$ from event $k$ to the moment immediately preceding the next event.

At each of these events ($k$ = 1, 2, $\ldots$, $C$-1) at which the population size changes, we allow the number of *births* $b_{k}$ (cell division) to be either 0 or 1, and the number of *deaths* $d_{k}$ (cells which leave the stem cell population, either via cell deaths, or via cell differentiation events) to any integer value from 0 up to $N_{k-1}$ (the size of the population when it enters event $k$).

Note that in a birth-death process it is more usual to assume that each event is either a birth event (where $b_{k}$ = 1, and $d_{k}$ = 0) or a death event (where $b_{k}$ = 0, and $d_{k}$ = 1). However, it turns out that while the analysis outlined below is greatly complicated if we allow $b_{k}$ to exceed 1, when we relax the constraints on $d_{k}$ we encounter very little additional difficulty. We have the following recursion for the population size

$$\begin{aligned} N_{k}=N_{k-1}+b_{k}-d_{k},\#\left( 1 \right) \end{aligned}$$

for $k$ = 1, 2, $\ldots$, $C$-1, subject to the constraints that either $b_{k}$ = 0 or $b_{k}$ = 1, and 0 $\leq d_{k}$ $\leq N_{k-1}$. There is one more sequence which it is useful for us to define here. This is the sequence of *drift intensities*, $\xi$ = $\left( \xi_{1},\xi_{2},\ldots,\xi_{C-1} \right)$, where

$\begin{aligned} \xi_{k}=\left( \frac{N_{k}}{2} \right)^{-1}b_{k}=\frac{2b_{k}}{N_{k}\left( N_{k}-1 \right)}\#\left( 2 \right) \end{aligned}$

for $k$ = 1, 2, $\ldots$, $C-1$. Recall that if there was no birth (cell division) at event $k$, then $b_{k}$ = 0, and therefore $\xi_{k}$ = 0. Notice that here we are using the conventional notation $\left( \frac{n}{k} \right)$, for binomial coefficients. In particular we have

$$\begin{aligned} \left( \frac{n}{2} \right)=\frac{n\left( n-1 \right)}{2}\#\left( 3 \right) \end{aligned}$$

We can define the function

$$b\left( t \right)=\sum_{k=1}^{C-1} b_{k}\delta\left( t,t_{k} \right),$$

which represents the intensity of birth events. We can also define the function

$\begin{aligned} \xi\left( t \right)=\sum_{k=1}^{C-1} \xi_{k}\delta\left( t,t_{k} \right),\#\left( 4 \right) \end{aligned}$

which represents the intensity of random drift.

We can express the drift intensity function as

$\begin{aligned} \xi\left( t \right)=\left( \frac{N\left( t \right)}{2} \right)^{-1}b\left( t \right)=\frac{2b\left( t \right)}{N\left( t \right)\left( N\left( t \right)-1 \right)},\#\left( 5 \right) \end{aligned}$

which is in agreement with the earlier definition (Equation 4). The trajectory of the intensity of random drift, as specified by the drift intensity function $\xi\left( t \right)$ (Equations 4 and 5), takes us a step closer to our goal of deriving an expression for the likelihood function for the sample phylogeny data. However, in order to express the likelihood function in its most familiar and convenient form, we need to express the trajectory $\xi\left( t \right)$ (and the related trajectories $N\left( t \right)$, and so on) as functions of time $s$ measured backwards from the time point at which the sample was collected ($s$ = 0). The relationship between the forward time $t$ (age from conception) and the backwards time $s$, is given by

$$s=t_{C}-t,$$

and hence $t$ = $t_{C}-s$.

So we can represent the backwards time trajectory for population size as the function

$$\tilde{N}\left( s \right)=N\left( t_{C}-s \right).$$

Similarly, we can represent the backwards time trajectories for other quantities of interest as follows

$$\tilde{b}\left( s \right)=b\left( t_{C}-s \right)$$

and

$$\tilde{\xi}\left( s \right)=\xi\left( t_{C}-s \right).$$

Now that we have this definition of the (reverse time) population size function $\tilde{N}\left( s \right)$, we can express the (reverse time) drift intensity function as

$\begin{aligned} \tilde{\xi}\left( s \right)=\left( \frac{\tilde{N}\left( s \right)}{2} \right)^{-1}\tilde{b}\left( s \right)=\frac{2\tilde{b}\left( s \right)}{\tilde{N}\left( s \right)\left( \tilde{N}\left( s \right)-1 \right)},\#\left( 6 \right) \end{aligned}$

which is simply the reverse time version of Equation 5.

Recall that we defined the sequence $t$ of distinct (forward) times (ages) at which the population size changes, together with the age $t_{C}$ at the time of sample collection, $t$ = $\left( t_{1},t_{2},\ldots,t_{C} \right)$, where 0 $<t_{1}$ $<t_{2}$ $<\ldots$ $<t_{C}$. The same sequence of time points, representing population events, which we have labelled with forward times (ages) $t_{k}$, can also be labelled with reverse times $s_{k}$ = $t_{C}-t_{k}$, for $k$ = 1, 2, $\ldots$, $C$-1. We now define the sequence $s$ of distinct reverse times at which the population size changes, together with the time $s_{0}$ (= $t_{C}$) at which conception occurred, $s$ = $\left( s_{0},s_{1},s_{2},\ldots,s_{C-1} \right)$, where $s_{k}$ = $t_{C}-t_{k}$, for each event $k$. Therefore, we have $s_{0}$ $>s_{1}$ $>s_{2}$ $>\ldots$ $>s_{C-1}$ $>0$. The function $\tilde{\xi}\left( s \right)$ (and the function $\xi\left( t \right)$) is completely determined by the sequence pair $\left( t,\xi\right)$, and also by the (equivalent) sequence pair $\left( s,\xi\right)$.

When the phylogeny, with (estimated) absolute branch lengths, is the only available data, the likelihood function of the model parameter given the data, is (up to a constant factor) equal to the joint probability density

$\begin{aligned} p_{n}\left( T\left( n \right),T\left( n-1 \right),\ldots,T\left( 2 \right);s,\xi\right)=\prod_{r=2}^{n} f_{r}\left( T\left( r \right)|S\left( r+1 \right);s,\xi\right),\#\left( 7 \right) \end{aligned}$

where

$$S\left( r \right)=T\left( n \right)+T\left( n-1 \right)+\ldots+T\left( r \right),$$

and where each factor

$\begin{aligned} f_{r}\left( w|s;s,\xi\right)=\left( \frac{r}{2} \right)\tilde{\xi}\left( s+w \right)\cdot R_{r}\left( w|s;s,\xi\right),\#\left( 8 \right) \end{aligned}$

is the (marginal) probability density of the waiting time to the next coalescent event, starting from time point $s$, when $r$ lines of descent remain (each of which can be traced back from the sample). The function $\tilde{\xi}\left( s \right)$ is the drift intensity at time $s$ (measured backwards from the time of sample collection). The function

$R_{r}\left( w|s;s,\xi\right)=exp\left[ -\left( \frac{r}{2} \right)\int_{u=s}^{u=s+w} \tilde{\xi}\left( u \right)du \right],$ (9)

gives the probability that the waiting time to the next coalescent event (starting from time point $s$, when $r$ lines of descent remain) is exceeds $w$. We could describe $R_{r}\left( w|s;s,\xi\right)$ as the *reliability* function (or survival function), and interpret $T\left( r \right)$ as a kind of *failure time* (at which one line of descent fails to persist).

Strictly speaking, Equations 8 and 9 represent an approximation which is valid whenever the entire sample phylogeny lies within a time interval throughout which the intensity of random drift $\tilde{\xi}\left( s \right)$ remains small (the effective population size remains large). See refs. ^80,81^ for derivation of the properties of the (reverse-time) genealogical process.

We want to draw attention to a feature of the likelihood function represented by Equations 7, 8, and 9. From the likelihood function (Equations 7 and 8) it is evident that, while segments (spanning certain time intervals) of the trajectory for the drift intensity (represented variously as a sequence pair $s$, $\xi$, or as a function of time), may constitute an identifiable parameter (when we have a phylogeny on a large enough sample), the trajectory for the population size, and the trajectory for the intensity of birth events, in the absence of additional constraints, are non-identifiable parameters. This is because the population sizes and the counts of birth events do not appear separately in the likelihood function, but only in the particular combination represented by the trajectory for the drift intensity.

In Section 3 below, parameter identifiability is defined more carefully, with some pointers to the literature. We also discuss in more detail the implications of non-identifiability for parameter estimation in our current model. In particular we will discuss how additional constraints on the population trajectory can restore identifiability of the population size, and the intensity of birth events.

**Parameter estimation and identifiability**

We usually make some further assumptions about the possible trajectories which the population is allowed to follow through time. In the case of a deterministic growth model, we assume that the sequence pair $\left( s,\xi\right)$ of event times and drift intensities belongs to a family of trajectories, in which the individual trajectory is completely determined by a parameter vector $\phi$. (Typically this parameter vector is of low dimension.) We say that the family of trajectories is parametrised by $\phi$. Here we have in mind models of deterministic exponential growth, where the model parameters include rates of cell division and rates of cell death.

In the case of a stochastic growth model, we assume that the sequence pair $\left( s,\xi\right)$ is drawn from a distribution which belongs to some family of distributions. Within this family of distributions, the specific distribution is completely determined by a parameter vector $\phi$. We say that the family of distributions is parametised by $\phi$. Here we have in mind models based on a birth death process, where the model parameters again include rates of cell division and rates of cell death.

In order to emphasise the dependence on the parameter vector $\phi$, it is convenient to use the notation

$$L\left( \phi|T \right) =p_{n}\left( T\left( n \right),T\left( n-1 \right),\ldots,T\left( 2 \right);\phi\right)$$

$=\prod_{r=2}^{n} f_{r}\left( T\left( r \right)|S\left( r+1 \right);\phi\right),$ (10)

for the likelihood function specified by Equations 7 and 8.

We say that a parameter vector $\phi$ is *non-identifiable* whenever there is a mapping $\vartheta$ (to a vector of lower dimension) for which the likelihood function $L\left( \phi|T \right)$ depends on the parameter vector $\phi$ only through $\theta$ = $\vartheta\left( \phi\right)$. In other words $\vartheta\left( \phi_{1} \right)$ = $\vartheta\left( \phi_{2} \right)$ implies that $L\left( \phi_{1}|T \right)$ = $L\left( \phi_{2}|T \right)$. If there is no such mapping $\vartheta$, then we say that the parameter vector $\phi$ is *identifiable*. When the parameter vector $\phi$ is *identifiable*, we may also refer to the components of this vector as *identifiable* parameters. See ref. ^82^ (*non-identifiability* is introduced in Section 3.15, on page 70, and discussed further on pages 72 and 74).

If such a mapping $\vartheta$ (to a vector of lower dimension) exists (so that $\phi$ is non-identifiable), then this means (loosely speaking) that from the fixed data $T$, we can not learn anything about the unobserved parameter vector $\phi$, beyond what we can learn about the (lower dimensional) parameter vector $\theta$. We can state this more precisely. First, we can always (leaving aside technical issues and pathological cases) express the prior density $\pi\left( \phi\right)$ for the parameter vector $\phi$, in the form

$\pi\left( \phi\right)=\pi\left( \phi|\theta\right)\pi\left( \theta\right).$ (11)

If $\phi$ is non-identifiable, and $\theta$ = $\vartheta\left( \phi\right)$ is identifiable, then the posterior density $\pi\left( \phi|T \right)$ of the parameter vector $\phi$ is of the form

$\pi\left( \phi|T \right)=\pi\left( \phi|\theta\right)\pi\left( \theta|T \right).$ (12)

As a consequence, we also have

$\pi\left( \phi|T,\theta\right)=\pi\left( \phi|\theta\right).$ (13)

This means that if we knew the (lower dimensional) parameter vector $\theta$, then the observed data $T$ would tell us nothing more about the (higher dimensional) parameter vector $\phi$.

First we consider a family of models where the population trajectory includes prolonged epochs during which birth events and death events occur equally often, so that the population size remains stable. Then we consider neutral models where the trajectory includes epochs of (deterministic) exponential population growth (Section 5). Finally, we consider birth-death processes, without an upper boundary (Section 6), and with an upper boundary (Section 7) on the population size, and how these stochastic growth models can be approximated by deterministic growth models.

**Epochs of stable effective population size**

First we consider a family of models where the population trajectory includes prolonged epochs during which the population size remains stable. Suppose that across the time interval $\left[ a,b \right]$, the population size remains constant at $N_{A}$. In order to maintain a constant population size, the birth rate $\beta_{A}$ must be balanced by an equal death rate.

The observed inter-coalescent interval durations $T\left( r \right)$, which fall within the time interval $\left[ a,b \right]$, contribute factors to the likelihood function which are of the form

$f_{r}\left( T\left( r \right)|S\left( r+1 \right);\phi\right)=\left( \frac{r}{2} \right)\frac{2\beta_{A}}{N_{A}}\cdot exp\left[ -\left( \frac{r}{2} \right)\frac{2\beta_{A}}{N_{A}}T\left( r \right) \right],$ (14)

where $\phi$ = $\left( N_{A},\beta_{A} \right)$ is the parameter vector of the model.

From the expression on the right-hand side of Equation 14, it appears that the only identifiable parameter is the ratio $\beta_{A}/N_{A}$.

**Epochs of exponential population growth**

Now we turn to neutral models where the trajectory includes epochs of exponential population growth. Suppose that the (forward time) estimated trajectory $\hat{\xi}\left( t \right)$ of the drift intensity appears to fit an exponential growth path across the time interval $\left[ t_{A},t_{C} \right]$, where $t_{C}$ is the time (age) at which the sample of $n$ genome-sequenced cells was collected. The estimated trajectory $\hat{\xi}\left( t \right)$ at time $t$ can be interpreted as a kind of average drift intensity over some interval centred on the time point $t$. The (forward time) estimated trajectory is

$\hat{\xi}\left( t \right)=\hat{k}\cdot exp\left[ \hat{\rho}\left( t-t_{A} \right) \right],$ (15)

which is based on point estimates $\hat{\rho}$ (for the growth rate) and $\hat{k}$ (for the initial drift intensity). Notice that when $\hat{\rho}$ is positive, the drift intensity declines exponentially, with increasing age $t$.

If we measure time backwards from sample collection, then the (reverse time) estimated trajectory $\hat{\tilde{\xi}}\left( s \right)$ of the drift intensity appears to fit an exponential growth path across the time interval $\left[ 0,s_{A} \right]$. The (reverse time) estimated trajectory is

$\hat{\tilde{\xi}}\left( s \right)=\hat{k}\cdot exp\left[ \hat{\rho}\left( s_{A}-s \right) \right],$ (16)

where $s_{A}$ = $t_{C}-t_{A}$ is the time measured backwards from sample collection to the time point at which the epoch of exponential growth began. Notice that when $\hat{\rho}$ is positive, the drift intensity increases exponentially, with increasing time $s$.

There is this one very simple model of population growth, in which births occur at a constant rate $\lambda$, and deaths occur at a constant rate $\nu$, which results in an exponential trajectory. This is an exceptionally parsimonious explanation for the observed exponential trajectory. If we can accept this parsimonious explanation, then we can set aside the general problem of making inferences about an arbitrary trajectory $\xi\left( t \right)$ for the intensity of random drift (the reciprocal of the effective population size), and restrict our attention to the very specific problem of making inferences about the parameters of the deterministic exponential growth model, or the parameters of the birth death process.

Having observed an (approximately) exponential trajectory for the drift intensity (and its reciprocal, the effective population size), from age $t_{A}$, up to the point of sample collection (at age $t_{C}$), we have arrived at a parsimonious explanation which we now examine in more detail. The population size has been growing at a constant growth rate $\rho$, while the birth rate has remained constant at a value $\lambda$, and the death rate has remained constant at a value $\nu$, which yields the constant growth rate $\rho$ = $\lambda-\nu$. Now we can express the trajectory for the population size $N\left( t \right)$, forward in time across the epoch of exponential growth (from age $t_{A}$ to age $t_{C}$) as

$N\left( t \right)=N_{A}exp\left[ \rho\left( t-t_{A} \right) \right],$ (17)

while the forward time trajectory for the drift intensity is

$\xi\left( t \right)=\frac{2\lambda}{N_{A}}\cdot exp\left[ -\rho\left( t-t_{A} \right) \right],$ (18)

where $N_{A}$ is the size of the ancestral population at age $t_{A}$ (when the epoch of exponential growth begins).

We now return to time measured backwards from sample collection. The reverse time trajectory for the population size is

$\tilde{N}\left( s \right)=N_{A}exp\left[ \rho\left( s_{A}-s \right) \right],$ (19)

where $s_{A}$ = $t_{C}-t_{A}$ is the time measured backwards from sample collection to the time point at which the epoch of exponential growth began. The reverse time trajectory for the drift intensity is

$\tilde{\xi}\left( s \right)=\frac{2\lambda}{N_{A}}\cdot exp\left[ -\rho\left( s_{A}-s \right) \right].$ (20)

The (marginal) probability density $f_{r}\left( w|s;\phi\right)$ of the waiting time to the next coalescent event (starting from time point $s$, when $r$ lines of descent remain), is in this case

$f_{r}\left( w|s;\phi\right) = \left( \frac{r}{2} \right)\frac{2\lambda}{N_{A}}\cdot exp\left[ \rho\left( w+s-s_{A} \right) \right]\cdot R_{r}\left( w|s;\phi\right),$ (21)

where $\phi$ = $\left( \lambda,\nu,N_{A} \right)$ is the parameter vector of this model, and where

$R_{r}\left( w|s;\phi\right) = exp\left[ -\left( \frac{r}{2} \right)\frac{2\lambda}{N_{A}}\cdot\frac{1}{\rho}exp\left[ \rho\left( s-s_{A} \right) \right]\left( e^{\rho w}-1 \right) \right],$ (22)

is the reliability function.

The observed inter-coalescent interval durations $T\left( r \right)$, which fall within the time interval $\left[ 0,s_{A} \right]$ (the epoch of exponential growth), contribute factors to the likelihood function which are of the form

$f_{r}\left( T\left( r \right)|S\left( r+1 \right);\phi\right)$

$= \left( \frac{r}{2} \right)\frac{2\lambda}{N_{A}}\cdot e^{-\rho\left( U\left( r \right)-T\left( r \right) \right)}\cdot exp\left[ -\left( \frac{r}{2} \right)\frac{2\lambda}{N_{A}}\cdot\frac{1}{\rho}e^{-\rho U\left( r \right)}\left( e^{\rho T\left( r \right)}-1 \right) \right],$ (23)

where $U\left( r \right)$ = $s_{A}-S\left( r+1 \right)$.

The parameter vector of this model is $\phi$ = $\left( \lambda,\nu,N_{A} \right)$, where $\lambda$ is the birth rate, $\nu$ is the death rate, and $N_{A}$ is the size of the ancestral population at the start of the epoch of exponential growth. (This occurs at age $t_{A}$, which precedes sample collection by time interval of duration $s_{0}$ = $t_{C}-t_{A}$.) From the formula for this factor of the likelihood function, it appears that the parameter vector $\phi$ is *non-identifiable*, while the parameter vector $\theta$ = $\left( N_{A}/\lambda,\rho\right)$ is *identifiable*. The components of the parameter vector $\theta$ are the ratio $N_{A}/\lambda$, and the difference $\rho$ = $\lambda-\nu$ (the population growth rate).

In the special case where the epoch of exponential growth (at constant growth rate $\rho$) extends all the way back to the founding individual (zygote cell), we know $N_{A}$ = 1, and we know that (reverse) time $s_{A}$ = $s_{C}$ (age $t_{A}$ = 0) corresponds to the moment of conception. In this special case, the unobserved parameters $\lambda$ and $\nu$, are identifiable. More generally, if the population size at the beginning of the epoch of exponential growth $N_{A}$ is known with certainty, then the parameter vector $\theta$ = $\left( \lambda,\nu\right)$ is *identifiable*.

In the case of a sample of single cell genome sequences obtained from blood-derived colonies, from a mouse (or any species with similar HSC dynamics), the parameter $N_{A}$ is the size of the ancestral population of HSCs at age $t_{A}$ (when the epoch of exponential growth begins); or if the time $t_{A}$ is even earlier, then $N_{A}$ is the size of the population of embryonic cells existing at this time which are ancestral to the HSCs. Unfortunately we do not have direct observations of the ancestral HSC population size $N_{A}$ (at the age $t_{A}$ when the epoch of exponential growth begins).

However, we can place some bounds on the value of $N_{A}$. First of all there is an upper bound $M_{A}$, on $N_{A}$, which can be obtained from embryological observations. We know the approximate number of cells in the embryo at age $t_{A}$. If some differentiation has already occurred, we may be able to exclude some cell types as HSC ancestors, and thus perhaps obtain an upper bound $M_{A}$ which is somewhat lower than the average total number of cells in a mouse embryo at age $t_{A}$. Secondly, we have a lower bound on $N_{A}$, which we can obtain directly from the phylogeny. This the number of lines of descent $n_{A}$ present on the tree at time $t_{A}$.

**The linear birth-death process**

A linear birth-death process is a simple stochastic growth model in which birth events and death events occur at constant rates (birth rate $\lambda$ and death rate $\nu$) per individual (cell) per unit of time (day or year). Therefore the total rate of birth (respectively death) events in the population at each time point is proportional to the total number of individuals in the population at that time point (hence a *linear* birth-death process. The total size $N\left( t \right)$ of the population at each time point is determined by the (stochastic) sequence of events (births and deaths) up to that time point. For the properties of the linear birth-death process, see ref. ^58^, and ref ^83^, pages 174–177.

Whenever the population size is not too small, and the growth rate is not too close to zero, the linear birth-death process behaves much like deterministic exponential growth. The trajectory for the population size $N\left( t \right)$ is well approximated by Equation 17, with growth rate $\rho$ = $\lambda-\nu$, provided that the birth rate $\lambda$ exceeds the death rate $\nu$, so that $\rho$ is positive.

In the case of an epoch of stochastic growth (under a linear birth-death process) it is important to bear in mind that the formula for the factors of the likelihood function in Equation 21, is an approximation, which can break-down. A conclusive argument about the identifiability of the model parameters should be based on an exact formula for the likelihood function for the linear birth-death process, when the phylogeny is the only available data.

**The birth-death process with an upper boundary on population size**

If a mouse lives long enough, we would expect that the propensity of the mouse HSC population to grow exponentially will eventually be checked by the physical constraints on the space available to accommodate the HSC cells within the bone marrow.

In the case of a model where the population undergoes deterministic exponential growth until an upper boundary $N_{B}$ on population size is reached, the phylogeny may contain additional information about the time $T_{B}$ at which the population first hits the upper boundary $N_{B}$. Such information can be present only if the sample of cells has been collected from the population at a time point after the time $T_{B}$. In this case, the hitting time parameter $T_{B}$ occurs in the likelihood function.

In the case of a model where the population undergoes deterministic exponential growth until an upper boundary $N_{B}$ population size is reached. The hitting time $T_{B}$ is determined by model parameters ($N_{A}/N_{B}$ and $\rho$ = $\lambda-\nu$). Using Equation 17, we can obtain

$\frac{N_{B}}{N_{A}}=exp\left[ \rho\left( T_{B}-t_{A} \right) \right],$ (24)

and therefore

$T_{B}=t_{A}+\frac{1}{\rho}ln\left( \frac{N_{B}}{N_{A}} \right).$ (25)

When the population reaches the upper boundary on population size, the marginal birth rate and the marginal death rate must be equal ($\delta_{B}$ = $\beta_{B}$). The parameter vector of the model is now $\phi$ = $\left( \lambda,\nu,N_{A},N_{B},\beta_{B} \right)$.

As usual we inspect the formula for the likelihood function in order to discover which parameters may be identifiable, and which are clearly non-identifiable. The factors of the likelihood function representing the epoch of exponential growth are of the form given in Equation 21, in which the parameter combinations $\lambda/N_{A}$ and $\rho$ appear. The factors of the likelihood function representing the epoch of stable population size are of the form given in Equation 14, in which the parameter combination $\beta_{B}/N_{B}$ appears. We have also seen from Equation 24 that the ratio $N_{B}/N_{A}$ is determined by the parameter $\rho$ and the the hitting time $T_{B}$. The hitting time $T_{B}$ is a change point, which also appears in the likelihood function. Therefore, from the formulas for the factors of the likelihood function, it appears that the parameter vector $\theta$ = $\left( \rho,\lambda/N_{A},\beta_{B}/N_{B},N_{B}/N_{A} \right)$ is identifiable. Notice also that by combining the last three components of $\theta$, we obtain

$$\frac{N_{B}}{N_{A}}\cdot\frac{\xi_{B}}{\xi_{A}}=\frac{N_{B}}{N_{A}}\cdot\frac{\beta_{B}}{N_{B}}\cdot\frac{N_{A}}{\lambda}=\frac{\beta_{B}}{\lambda}.$$

So the ratio $\beta_{B}/\lambda$ is also identifiable.

In the special case where $N_{A}$ is known for certain, the parameter vector $\theta$ = $\left( \lambda,\nu,N_{B},\beta_{B} \right)$ is identifiable. As already discussed in Section 5, when the epoch of exponential growth (at constant growth rate $\rho$) extends all the way back to the founding individual (zygote cell), we know $N_{A}$ = 1. So, in this case, the parameters $\lambda$, $\nu$, $N_{B}$, and $\beta_{B}$, are all identifiable, and amenable to estimation from the phylogeny of a sample.

# Supplementary note references

75. Flurkey, K., M. Currer, J. & Harrison, D. E. Mouse Models in Aging Research. in *The Mouse in Biomedical Research (Second Edition)* (eds. Fox, J. G. et al.) 637–672 (Academic Press, Burlington, 2007). doi:10.1016/B978-012369454-6/50074-1.

76. Pritchard, J. K., Seielstad, M. T., Perez-Lezaun, A. & Feldman, M. W. Population growth of human Y chromosomes: a study of Y chromosome microsatellites. *Molecular Biology and Evolution* **16**, 1791–1798 (1999).

77. Csilléry, K., François, O. & Blum, M. G. abc: an R package for approximate Bayesian computation (ABC). *Methods in ecology and evolution* **3**, 475–479 (2012).

78. Lan, S., Palacios, J. A., Karcher, M., Minin, V. N. & Shahbaba, B. An efficient Bayesian inference framework for coalescent-based nonparametric phylodynamics. *Bioinformatics* **31**, 3282–3289 (2015).

79. Moran, P. A. P. Random processes in genetics. *Proceedings of the Cambridge Philosophical Society* **54**, 60–71 (1958).

80. Kingman, J. F. C. On the Genealogy of Large Populations. *J. Appl. Probab.* **19A**, 27–43 (1982).

81. Griffiths, R. C. & Tavaré, S. Sampling theory for neutral alleles in a varying environment. *Philosophical Transactions of the Royal Society, London, Series B* **344**, 403–410 (1994).

82. O’Hagan, A. & Forster, J. *Bayesian Inference*. vol. 2B (Arnold, London, UK, 2004).

83. Moran, P. A. P. *An Introduction to Probability Theory*. (Oxford University Press, Oxford, UK, 1968).
