# Supplementary material for: Clonal dynamics and somatic evolution of haematopoiesis in mouse
Source: Nature. 2025 Mar 5;641(8063):681–9. doi: 10.1038/s41586-025-08625-8 (PMC12074984; doi:10.1038/s41586-025-08625-8)
Supplement: Supplementary file 2 — Reporting Summary [file 41586_2025_8625_MOESM2_ESM.pdf]

nature portfolio

Corresponding author(s): Margaret A. Goodell & Jyoti Nangalia

Last updated by author(s): Dec 18, 2024

Reporting Summary

Nature Portfolio wishes to improve the reproducibility of the work that we publish. This form provides structure for consistency and transparency in reporting. For further information on Nature Portfolio policies, see our Editorial Policies and the Editorial Policy Checklist.

Statistics

For all statistical analyses, confirm that the following items are present in the figure legend, table legend, main text, or Methods section.

n/a	Confirmed	
☐	☒	The exact sample size (*n*) for each experimental group/condition, given as a discrete number and unit of measurement
☐	☒	A statement on whether measurements were taken from distinct samples or whether the same sample was measured repeatedly
☐	☒	The statistical test(s) used AND whether they are one- or two-sided<br>*Only common tests should be described solely by name; describe more complex techniques in the Methods section.*
☒	☐	A description of all covariates tested
☐	☒	A description of any assumptions or corrections, such as tests of normality and adjustment for multiple comparisons
☐	☒	A full description of the statistical parameters including central tendency (e.g. means) or other basic estimates (e.g. regression coefficient) AND variation (e.g. standard deviation) or associated estimates of uncertainty (e.g. confidence intervals)
☐	☒	For null hypothesis testing, the test statistic (e.g. *F*, *t*, *r*) with confidence intervals, effect sizes, degrees of freedom and *P* value noted<br>*Give P values as exact values whenever suitable.*
☐	☒	For Bayesian analysis, information on the choice of priors and Markov chain Monte Carlo settings
☐	☒	For hierarchical and complex designs, identification of the appropriate level for tests and full reporting of outcomes
☐	☒	Estimates of effect sizes (e.g. Cohen's *d*, Pearson's *r*), indicating how they were calculated

Our web collection on statistics for biologists contains articles on many of the points above.

Software and code

Policy information about availability of computer code

Data collection

Flow cytometry data was collected using BD FACSDiva version 9.0 and analyzed using FlowJo version 10.8.1.

Data analysis

SNVs and indels were detected using CaVEMan (version 1.14.0, https://github.com/cancerit/CaVEMan), cgpPindel (version 3.9.0, https://github.com/cancerit/cgpPindel), and VarDict (version 1.8.3, https://github.com/AstraZeneca-NGS/VarDictJava). Variants were annotated using VAGrENT (version 3.7.0, https://github.com/cancerit/VAGrENT), and Ensemble VEP (release 107-110.0, https://github.com/Ensembl/ensembl-vep). Phylogenies were constructed using MPBoot (version 1.1.0, https://github.com/diepthihoang/mpboot). Variants were assigned to phylogenies using Rtreemut (https://github.com/nangalialab/treemut). Population trajectories were inferred using phylodyn (https://github.com/mdkarcher/phylodyn). Bayesian inferences utilized the packages rsimpop (https://github.com/nangalialab/rsimpop) for simulations and abc (version 2.2.1, https://CRAN.R-project.org/package=abc) for approximate Baysesian Computation. Mutation signatures were inferred using the hdp (https://github.com/nicolaroberts/hdp) and sigfit (version 2.2.0, https://github.com/kgori/sigfit). Duplex consensus reads were generated using the fgbio suite of tools (version 1.5.1-2.1.0, http://fulcrumgenomics.github.io/fgbio/). dN/dS ratios were calculated using dNdScv (version 0.1.0, https://github.com/im3sanger/dndscv). Custom DNA baitsets were designed using the Agilent SureDesign tool (version 7.10.2). Population genetic analyses of clone sizes and parameter inferences were based on code available at https://github.com/blundelllab/ClonalHematopoiesis/. Other analyses were carried out using custom R scripts (R version 4.3) and will be are available at https://github.com/CDKapadia/somatic-mouse.

For manuscripts utilizing custom algorithms or software that are central to the research but not yet described in published literature, software must be made available to editors and reviewers. We strongly encourage code deposition in a community repository (e.g. GitHub). See the Nature Portfolio guidelines for submitting code & software for further information.

Data

Policy information about availability of data

All manuscripts must include a data availability statement. This statement should provide the following information, where applicable:

- Accession codes, unique identifiers, or web links for publicly available datasets
- A description of any restrictions on data availability
- For clinical datasets or third party data, please ensure that the statement adheres to our policy

Whole genome sequencing data will be deposited at the European Nucleotide Archive at accession numbers ERP138320, ERP152795, and ERP144323. Targeted duplex sequencing data has been deposited at NCBI BioProject PRJNA1033340.

Research involving human participants, their data, or biological material

Policy information about studies with human participants or human data. See also policy information about sex, gender (identity/presentation), and sexual orientation and race, ethnicity and racism.

Reporting on sex and gender	No human data in this study.
Reporting on race, ethnicity, or other socially relevant groupings	No human data in this study.
Population characteristics	No human data in this study.
Recruitment	No human data in this study.
Ethics oversight	No human data in this study.

Note that full information on the approval of the study protocol must also be provided in the manuscript.

Field-specific reporting

Please select the one below that is the best fit for your research. If you are not sure, read the appropriate sections before making your selection.

☒ Life sciences ☐ Behavioural & social sciences ☐ Ecological, evolutionary & environmental sciences

For a reference copy of the document with all sections, see nature.com/documents/nr-reporting-summary-flat.pdf

Life sciences study design

All studies must disclose on these points even when the disclosure is negative.

Sample size	No power calculations were used to determine sample size. Sample size was determined based on feasible experimental size and subject survival.
Data exclusions	Whole-genome data was excluded due to quality control failures as described in the methods. Exact numbers of excluded data are described specifically.<br>-Some colonies were excluded based on low coverage or evidence of non-clonality or contamination. Visual inspection of filtered variant VAF distributions per colony was used to identify colonies with mean variant allele fraction (VAF)<0.4 or with evidence of non-clonality (Extended Data Fig. 1C).<br>-Following exclusion of 106 colonies due to low sequencing coverage or lack of clonality (Extended Data Fig.1C, methods), 1305 whole genomes (666 HSC, 639 MPP) were taken forward for somatic mutation identification and phylogenetic reconstruction.
Replication	Whole genome analysis was not replicated due to large per-subject datasets. For duplex experiments, each individual mouse queried is an independent biological replicate. In perturbation experiments, displayed data is aggregated from at least 2 independent replicate treatment cohorts. All subjects within experimental cohorts are included in the aggregated data. Replicate experiments were designed identically. All replicate experiments were successful.
Randomization	Mice were randomly allocated to control or experimental groups.
Blinding	Investigators were not blinded to the group assignment during experiments or during analysis because the same investigators performed all mouse experiments and subsequent bioinformatics analyses.

Reporting for specific materials, systems and methods

We require information from authors about some types of materials, experimental systems and methods used in many studies. Here, indicate whether each material, system or method listed is relevant to your study. If you are not sure if a list item applies to your research, read the appropriate section before selecting a response.

Materials & experimental systems

n/a	Involved in the study
☐	☒ Antibodies
☒	☐ Eukaryotic cell lines
☒	☐ Palaeontology and archaeology
☐	☒ Animals and other organisms
☒	☐ Clinical data
☒	☐ Dual use research of concern
☒	☐ Plants

Methods

n/a	Involved in the study
☒	☐ ChIP-seq
☐	☒ Flow cytometry
☒	☐ MRI-based neuroimaging

Antibodies

Antibodies used

• FACS: anti-CD150-Bright Violet 711 (Biolegend, catalog number 115941, clone TC15-12F12.2, 1:50)
• FACS: anti-CD48-FITC (eBioscience, catalog number 11-0481-82, clone HM48-1, 1:100)
• FACS: anti-CD117-APC (eBioscience, catalog number 17-1171-83, clone 2B8, 1:100)
• FACS: anti-FLT3-PE (eBioscience, catalog number 12-1351-82, clone A2F10, 1:50)
• FACS: anti-Sca1-PE-Cy7 (eBioscience, catalog number 25-5981-82, clone D7, 1:100)
• FACS: anti-Ter119-eFlour-450 (eBioscience, catalog number 48-5921-82, clone Ter119, 1:100)
• FACS: anti-Gr1-eFlour-450 (eBioscience, catalog number (48-5931-82, clone RB6-8C5, 1:100)
• FACS: anti-CD8a-eFlour-450 (Invitrogen, catalog number 48-0081-82, clone 53-6.7, 1:100)
• FACS: anti-Mac1-eFlour-450 (eBioscience, catalog number 48-0112-82, clone M1/70, 1:100)
• FACS: anti-CD4-eFlour-450 (eBioscience, catalog number 48-0041-82, clone GK1.9, 1:100)
• FACS: anti-B220-eFlour-450 (eBioscience, catalog number 48-0452-82, clone 48-0452-82., 1:100)

Validation

All antibodies are from commercial vendors and have been validated by the manufacturer with supporting publications found on the manufacturer's website for each antibody. See below for summary:

FACS: anti-CD150-Bright Violet 711 (Biolegend, catalog number 115941, clone TC15-12F12.2, 1:50)
Species: Mouse
Application: FACS

FACS: anti-CD48-FITC (eBioscience, catalog number 11-0481-82, clone HM48-1, 1:100)
Species: Mouse
Application: FACS

FACS: anti-CD117-APC (eBioscience, catalog number 17-1171-83, clone 2B8, 1:100)
Species: Mouse
Application: FACS

FACS: anti-FLT3-PE (eBioscience, catalog number 12-1351-82, clone A2F10, 1:50)
Species: Mouse
Application: FACS

FACS: anti-Sca1-PE-Cy7 (eBioscience, catalog number 25-5981-82, clone D7, 1:100)
Species: Mouse
Application: FACS

FACS: anti-Ter119-eFlour-450 (eBioscience, catalog number 48-5921-82, clone Ter119, 1:100)
Species: Mouse
Application: FACS

FACS: anti-Gr1-eFlour-450 (eBioscience, catalog number (48-5931-82, clone RB6-8C5, 1:100)
Species: Mouse
Application: FACS

FACS: anti-CD8a-eFlour-450 (Invitrogen, catalog number 48-0081-82, clone 53-6.7, 1:100)
Species: Mouse
Application: FACS

FACS: anti-Mac1-eFlour-450 (eBioscience, catalog number 48-0112-82, clone M1/70, 1:100)
Species: Mouse
Application: FACS

FACS: anti-CD4-eFlour-450 (eBioscience, catalog number 48-0041-82, clone GK1.9, 1:100)
Species: Mouse
Application: FACS

FACS: anti-B220-eFlour-450 (eBioscience, catalog number 48-0452-82, clone 48-0452-82., 1:100)
Species: Mouse
Application: FACS

Animals and other research organisms

Policy information about studies involving animals; ARRIVE guidelines recommended for reporting animal research, and Sex and Gender in Research

Laboratory animals	Mouse strain, age, and sex is described in the manuscript text, legends, and methods. All mice were C57BL/6, C57BL/6J:FVB/NJ F1 hybrid mice , or HET3 mice. Mouse age ranged from 3 months to 36 months, as described in the manuscript. Wild-type C57BL/6 mice were bred at Baylor College of Medicine or received from the Aged Rodent Colony at the National Institute of Aging (Baltimore, MD). C57BL/6J:FVB/NJ F1 hybrid mice were bred in the Niedernhofer laboratory at the University of Minnesota. HET3 mice were bred at the Jackson Laboratories. C57BL/6 were housed at the AAALAC-approved Center for Comparative Medicine in BSL-2 suites. Mice were housed with the same sex in ventilated cages, under a 14/10-hour light-dark cycle with temperature and humidity control, enrichment material, and fed ad libitum with a standard chow diet.
Wild animals	No wild animals were used in this study.
Reporting on sex	Duplex sequencing experiment used a mix of male and female mice because clonal hematopoiesis occurs in both sexes. For whole genome colony data, all mice were female.
Field-collected samples	No field collected samples were used for this study.
Ethics oversight	Experimental procedures were approved by the Baylor College of Medicine or University of Minnesota Institutional Animal Care and Use Committees and performed following the Office of Laboratory Animal Welfare guidelines and PHS Policy on Use of Laboratory Animals.

Note that full information on the approval of the study protocol must also be provided in the manuscript.

Plants

Seed stocks	No plants were used in this study.
Novel plant genotypes	No plants were used in this study.
Authentication	No plants were used in this study.

Flow Cytometry

Plots

Confirm that:

☒ The axis labels state the marker and fluorochrome used (e.g. CD4-FITC).

☒ The axis scales are clearly visible. Include numbers along axes only for bottom left plot of group (a 'group' is an analysis of identical markers).

☒ All plots are contour plots with outliers or pseudocolor plots.

☒ A numerical value for number of cells or percentage (with statistics) is provided.

Methodology

Sample preparation	Whole bone marrow (WBM) cells were isolated from murine hindlimbs and enriched for c-Kit+ hematopoietic progenitors prior to fluorescence-activated cell sorting (FACS). WBM was incubated with anti-CD117 microbeads (Miltenyi) for 30 minutes at 4C following my magnetic column enrichment (LS Columns, Miltenyi). HSCs were defined as Lineage–ckit+Sca-1 +FLT-3–CD48–CD150+; MPPs were defined as Lineage–ckit+Sca-1+FLT-3–CD48–CD150–. The gating strategy is illustrated in Extended Data Fig. 1A.
Instrument	FACS was completed on a BD FACSAriaII.
Software	Flow cytometry data was collected using BD FACSDiva version 9.0 and analyzed using FlowJo version 10.8.1.

Cell population abundance	Cell population abundance is described in Figure 3b and Extended Data 1A.
Gating strategy	Gating strategy is describing in Extended Data 1A.

☒ Tick this box to confirm that a figure exemplifying the gating strategy is provided in the Supplementary Information.
